# COG lobe B sub-complex engages v-SNARE GS15 and functions via regulated interaction with lobe A sub-complex

**DOI:** 10.1038/srep29139

**Published:** 2016-07-07

**Authors:** Rose Willett, Jessica Bailey Blackburn, Leslie Climer, Irina Pokrovskaya, Tetyana Kudlyk, Wei Wang, Vladimir Lupashin

**Affiliations:** 1 Department of Physiology and Biophysics, UAMS, Little Rock, AR, USA.

## Abstract

The conserved oligomeric Golgi (COG) complex is a peripheral membrane protein complex which orchestrates tethering of intra-Golgi vesicles. We found that COG1-4 (lobe A) and 5–8 (lobe B) protein assemblies are present as independent sub-complexes on cell membranes. Super-resolution microscopy demonstrates that COG sub-complexes are spatially separated on the Golgi with lobe A preferential localization on Golgi stacks and the presence of lobe B on vesicle-like structures, where it physically interacts with v-SNARE GS15. The localization and specific interaction of the COG sub-complexes with the components of vesicle tethering/fusion machinery suggests their different roles in the vesicle tethering cycle. We propose and test a novel model that employs association/disassociation of COG sub-complexes as a mechanism that directs vesicle tethering at Golgi membranes. We demonstrate that defective COG assembly or restriction of tethering complex disassembly by a covalent COG1-COG8 linkage is inhibitory to COG complex activity, supporting the model.

The vesicular transport pathway requires the concerted actions of both structural and regulatory protein families to orchestrate the formation, delivery, and fusion of a transport intermediate/vesicle to its acceptor compartment^1^. One family of these regulatory proteins is the multisubunit tethering complexes (MTC’s), which are believed to function in organizing, tethering, and subsequent fusion of transport vesicles with their target membrane via interactions with both target and vesicle membrane proteins. MTC’s are found throughout the entire secretory pathway, with different MTC’s guiding each step in the pathway. Furthermore, structural, subunit organization, and interactome similarities of the different MTC’s suggests that they may also function in a homologous manner^2^.

The major MTC which functions at the Golgi apparatus is the conserved oligomeric Golgi (COG) complex. The COG complex is a peripheral membrane protein complex that cycles between the cytosol and Golgi/vesicle membranes^3^,^4^,^5^,^6^,^7^. The COG complex is composed of eight subunits (named COG1-8), which are separated into two sub-complexes: lobe A (COGs 1–4) and lobe B (COGs 5–8)^3^,^8^, with an interaction between COG1 and COG8 bridging the two lobes together. The COG complex tethers vesicles recycling Golgi resident proteins (such as glycosylation enzymes) and therefore is essential for the proper glycosylation of secretory proteins^9^,^10^,^11^.

The bi-lobed model of the COG complex is a well-established depiction of the eight COG subunits. EM images of purified bovine COG have confirmed the bi-lobed organization^4^. Functional data of the COG complex suggests that this bi-lobed model might be an over-simplification of the possible arrangements of the COG complex subunits. It has been previously demonstrated that lobe A subunits are essential in yeast, whereas lobe B subunit deletions are functionally viable^5^,^12^, suggesting that lobe A and B subunits may perform separate trafficking functions. The phenotypic differences in lobe A and lobe B subunit mutations in some model organisms highlight the idea of a working separation between the sub-complexes. Furthermore, siRNA induced knockdown (KD) of lobe A subunits in HeLa cells results in drastic fragmentation of the Golgi apparatus whereas lobe B subunit KD’s have much milder effects on Golgi morphology^6^,^13^. Surprisingly, this was not the case in HEK293T cells completely depleted of individual COG subunits using a CRISPR/Cas9 strategy^14^. All knockout cell lines were uniformly deficient in cis/medial-Golgi glycosylation and showed pronounced defects in Golgi morphology. We hypothesize that working separation may also translate into a physical segregation of lobe A and lobe B sub-complexes. All previous studies of COG complex organization were based on the analysis of soluble purified COG complex^4^ while its major cellular function is tightly coupled to membranes and transmembrane proteins. Therefore, we sought to understand the arrangement(s) of COG subunits on membranes, both in steady-state and in living cells during the active membrane trafficking process.

In this work we set out to determine if COG sub-complexes, lobe A and lobe B, are stable membrane-bound arrangements of the COG complex *in vivo*. Using size exclusion chromatography and native immunoprecpitation (IP), we discovered that the membrane associated COG complex is largely separated to lobe A and lobe B tetramers. Furthermore, we use live cell and super-resolution microscopy to demonstrate that the majority of lobe A associates with Golgi membranes, while the majority of lobe B associates with vesicle membranes. COG sub-complex separation on different membranes is re-enforced by their specific interactions with distinct components of vesicle tethering/fusion machinery. Finally, we propose and test a model of COG complex-mediated vesicle tethering that utilizes the transient interaction between lobe A and lobe B sub-complexes on opposing membranes to facilitate vesicle tethering/organization at the Golgi apparatus. These results suggest a new detailed mechanistic model of COG complex mediated vesicle tethering at the Golgi apparatus.

## Results

### Membrane-associated COG sub-complexes exist independently

**Figure 1:**
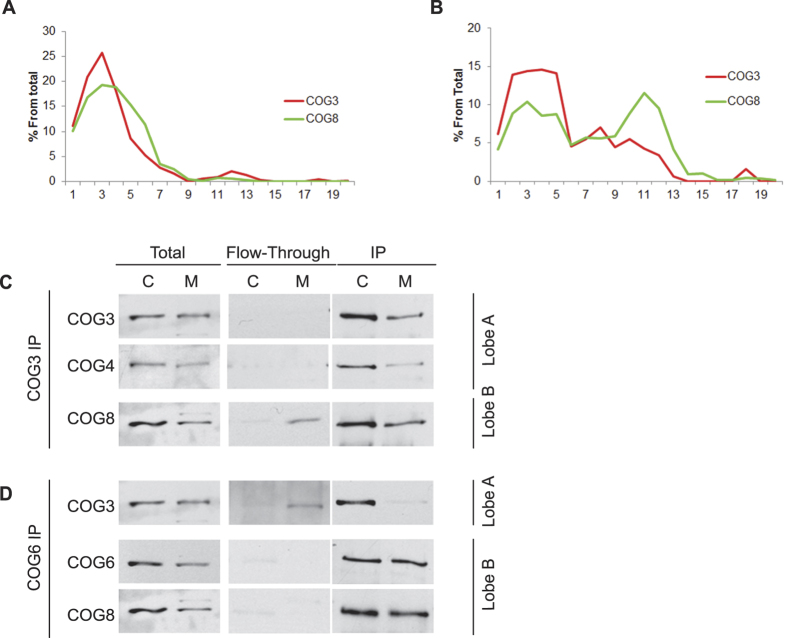
Membrane associated COG subunits are arranged as octamers and tetramers. HeLa cell lysates were separated into cytosol [C] and membrane [M] fractions by differential centrifugation. Efficient separations were confirmed by SDS-PAGE using antibodies against GAPDH (cytosolic enzyme) and Vti1a (transmembrane protein) (Supplemental Figure 1C). HeLa cytosol (**A**) or Triton-X100 soluble membranes (**B**) were loaded onto a Superose 6 10/300 GL and 0.5 mL fractions were collected. Fractions were concentrated by TCA precipitation, separated on a SDS-PAGE gel, transferred to a nitrocellulose membrane, and probed with anti-COG3 and anti-COG8 antibodies. Soluble COG subunits peaked in early fractions (fractions 2–4; Ve = ~9–10.5 mL) indicative of the size of the COG complex octamer. Membrane COG subunits peaked in fractions 2–4 (Ve = ~9–10.5 mL) and also in fractions 10–12 (Ve: ~12.5–14 mL) corresponding in size to a tetramer. HeLa cytosol and membrane fractions were immunoprecipitated (IP) with anti-COG3 (**C**) and anti-COG6 antibodies (**D**). Immunodepleted/flow-through (F/T) and IP fractions were separated by SDS-PAGE and blotted with antibodies to COG3, COG4, COG6, and COG8. IP of lobe A subunit COG3 immunodepleted lobe B subunit COG8 from cytosol, but not membrane fraction. IP of lobe B subunit immunodepleted lobe A subunits COG3 from cytosol, but not membrane fraction.

To gain a better understanding of the major molecular arrangements of the COG subunits *in vivo,* we performed a gel filtration analysis of the endogenous COG proteins present in both cytosol and membrane fractions isolated from HeLa cells. In this analysis we used two evolutionary conserved subunits from both lobes of the COG complex. Distribution of endogenous COG3, COG4, COG6 and COG8 were detected by Western blot (WB). In accordance with previously published data^6^, we detected a 60:40 split of the COG subunits in cytosolic and membrane fractions, respectively, upon cell disruption and differential centrifugation (Fig. 1C). Triton X-100 solubilized proteins from both fractions were separated over a Superose 6 size exclusion column and analyzed for individual COG subunits by WB. The fractionation volume was then matched to a standardized curve to estimate the eluted protein(s) size (Supplemental Figure 1A,B). We observed that in cytosolic fractions, more than 90% of both lobe A subunit COG3 and lobe B subunit COG8 co-eluted in early fractions 2–6, corresponding in size to the octameric complex (Fig. 1A). Similar results were obtained for COG5 and COG6 (Supplemental Figure 1D,E), indicating that the bulk of cytosolic COG subunits exist as an octamer in HeLa cells. Small amounts of both COG6 (~15–25%) and COG5 (~10–20%), but not COG3 or COG8, were also present in fractions 12–13, corresponding to the size of a hypothetical COG5/6/7 trimer (~250 kDa)^15^. The soluble COG5/6/7 trimer could exist in the cytosol to perform a yet unknown specialized function, or alternatively, could be an artifact of partial COG dissociation during the gel-filtration procedure. However, none of the subunits were detected in fractions 16–18 corresponding to monomeric subunits, confirming that the majority of the soluble COG complex is restricted to the octamer arrangement and that the COG complex is not significantly fragmented during the gel-filtration process. These results are in agreement with previous fractionation of the COG complex from total bovine brain^4^ and from CHO cell whole lysate^16^ where lobe A and lobe B subunits can be detected co-fractionating as a large protein complex. Detergent extracted membrane material separated over the same column gave a different profile of the eluted COG proteins. In addition to the elution peak in fractions 2–4 (Ve: ~9–10.5 mL), lobe A subunit COG3 and lobe B subunit COG8 also co-eluted in a second peak in fractions 10–12 (Ve: ~12.5–14 mL), corresponding in size to a tetramer (Fig. 1B). Interestingly, almost 50% of membrane-associated COG5, COG6, and COG8 were found associated with this smaller sub-complex. Again, like in the cytosolic separation, we did not observe any COG subunits in fractions corresponding to a monomeric protein.

Gel filtration results indicated that a significant portion of the membrane-associated COG subunits exist as stable sub-complexes or in a stable assembly with other unknown membrane-bound proteins. We verified this separation of COG sub-complexes by native immunoprecipitation (IP). Endogenous COG subunits were IP’d from either cytosolic or membrane fractions with antibodies directed against lobe A subunit COG3 or lobe B subunit COG6, and then both unbound (flow-through, FT) and IP material were subjected to WB analysis. In this experiment we utilized available antibodies for two proteins from each COG sub-complexes. The recovery of individual COG subunits in the IP fraction was always less than 100% probably due to intense multiple washes of the immunoprecipitates, so we have concentrated our analysis on the co-depletion of individual COG subunits in FT fractions. Affinity-purified anti-COG3 and anti-COG6 antibodies were efficient in removing all specific antigens from FT material, indicating that IPs were complete (Fig. 1C,D Flow-through columns). IP of COG3 from the cytosol and membrane fractions resulted in co-IP of both lobe A and lobe B subunits (Fig. 1C,D, IP fractions), confirming that all tested COG subunits are associated in a stable detergent-resistant COG1-8 octamer. Depletion of COG3 in FT material also resulted in the complete depletion of COG4 in both cytosol and membrane fractions, indicating that COG3 and COG4 (and by extension two other lobe A subunits COG1 and COG2) are stably associated in a complex/sub-complex in both soluble and membrane-associated pools (Fig. 1C COG4, FT column). COG8 was completely co-depleted with lobe A subunits from cytosolic fractions, but only partially depleted from the membrane fractions (Fig. 1C FT), indicating that ~50% of membrane-bound COG8 is not associated with lobe A subunits. Similar results were obtained in COG6 native IP (Fig. 1D). Again, depletion of COG6 in FT material coincided with the complete depletion of COG8 in both cytosol and membrane fractions, indicating that COG6 and COG8 (and by extension two other lobe B subunits COG5 and COG7) are stably associated in a complex/sub-complex in both soluble and membrane-associated pools. Likewise COG3 was completely co-depleted with lobe B subunits from cytosol, but only partially from a membrane fraction, indicating that ~50% of membrane-bound COG3 is not associated with lobe B subunits. Based on these results, we conclude that the smaller complexes identified by gel filtration are indeed the lobe A and lobe B sub-complexes. Combining the native IP and gel filtration data, we conclude that lobe A and lobe B are tightly associated with one another in the soluble pool of COG, whereas the membrane-associated COG subunits exist as a combination of the complete COG1-8 octamer, as well as lobe A and lobe B sub-complexes.

**Figure 2:**
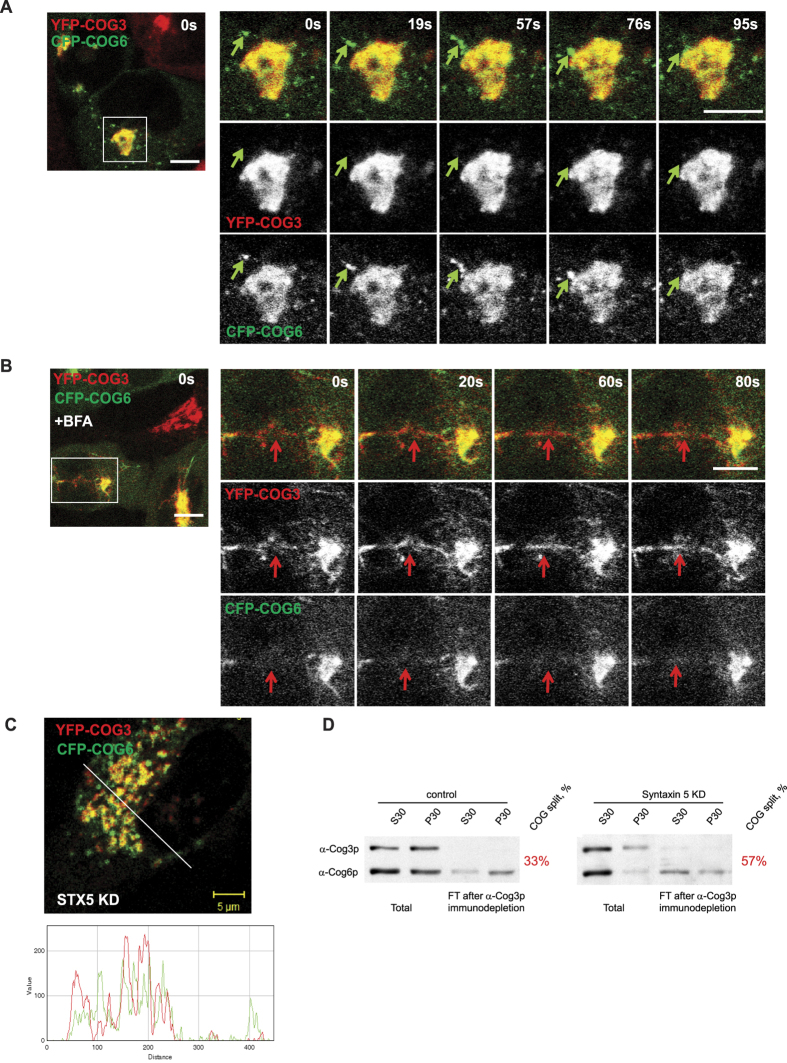
COG sub-complex lobe B localizes to Golgi and vesicle membranes. (**A**) Static live cell images of HeLa cells stably expressing lobe A subunit YFP-COG3 (red) and lobe B subunit CFP-COG6 (green). CFP-COG6 partially localizes to smaller vesicle-like structures separate from YFP-COG3 and Golgi apparatus (**A**). Green arrows indicate structure labeled with CFP-COG6 but not YFP-COG3 moving towards and fusing with the Golgi. (**B**) Static live images of HeLa YFP-COG3/CFP-COG6 cells treated with 0.25 μg/l BFA for 15 min. Red arrow indicates a YFP-COG3 labeled tubule which does not contain CFP-COG6. Bar, 10 μm. (**C**) HeLa YFP-COG3/CFP-COG6 cells depleted for Golgi t-SNARE STX5 were fixed and analyzed by IF. Line plot for overlap between red and green channels is shown measuring the relative value of signal intensity (y-axis) over the distance measured in pixels (x-axis). Bar, 5 μm. (**D**) HeLa control and STX5 depleted cells were separated by centrifugation on S30 and P30 fractions and immunodepleted using anti-COG3 antibodies. Initial and immunodepleted fractions were separated by SDS-PAGE and blotted with antibodies against COG3 and COG6. Numbers indicate percentage of lobe B COG6 protein not associated with lobe A COG3.

Our biochemical results indicate that membrane-associated COG proteins are in either a static or dynamic arrangement of the COG sub-complexes. Therefore, we sought to visualize this separation on Golgi/vesicle membranes using a combination of live cell imaging and super-resolution microscopy. HeLa cells stably expressing YFP-COG3 and CFP-COG6 (at near endogenous levels, Supplemental Figure 2) were visualized to detect the dynamics of lobe A and lobe B subunits *in vivo*. Both fluorescent signals were primarily found associated with the perinuclear Golgi area (Fig. 2A). Live imaging revealed that a sub-population of CFP-COG6 was also present on rapidly moving vesicle-like structures. CFP-COG6 positive vesicle-like structures were observed in 80% of YFP-COG3/CFP-COG6 cells (n = 40). In the remaining 20% of cells, expression of CFP-COG6 was not detected by confocal microscopy. Several of these COG6-positive vesicle-like structures were captured moving toward the Golgi area and fusing with larger COG3-positive membranes (Fig. 2A, time frames; green arrow indicate COG6-positive vesicle-like structures). Similar lobe B-positive vesicle-like structures were observed in cells expressing COG5-GFP and COG8-GFP (data not shown). Interestingly, none of these smaller structures exclusively labeled with YFP-COG3 were detected, suggesting Golgi (large membrane) localization of membrane-bound lobe A. To untangle the Golgi structure, we treated cells with a low concentration (0.25 μM) of Brefeldin A^9^. Ten minutes after BFA treatment, Golgi tubulation was observed with many membrane extensions preferentially occupied by either YFP-COG3 or CFP-COG6, (Fig. 2B, time frames; red arrow show YFP-COG3-positive tubules) suggesting the existence of separate lobe A subcomplex on Golgi membranes. We have shown previously that functional COG complex is required for the formation and/or stability of the intra-Golgi STX5-containinig SNARE complex^17^. We have reasoned here that if COG sub-complex arrangement is dynamic it could be regulated by Golgi SNARE machinery. Indeed, downregulation of STX5 in HeLa cells stably expressing YFP-COG3 and CFP-COG6 leads to Golgi fragmentation and more evident spatial separation of membranes preferentially occupied by either COG3 or COG6 (Fig. 2C and Supplemental Figure 2B). Moreover, biochemical fractionation of HeLa STX5 KD cells into P30 (Golgi-enriched fraction) and S30 (soluble and vesicle fraction) revealed substantial depletion of the endogenous COG6 in the Golgi fraction and corresponding decreased in interaction between COG3 and COG6 (Fig. 2D). These results indicate that functional Golgi SNARE machinery (productive vesicle fusion machinery) is necessary for the normal balance between the assembled and disassembled COG arrangements.

**Figure 3:**
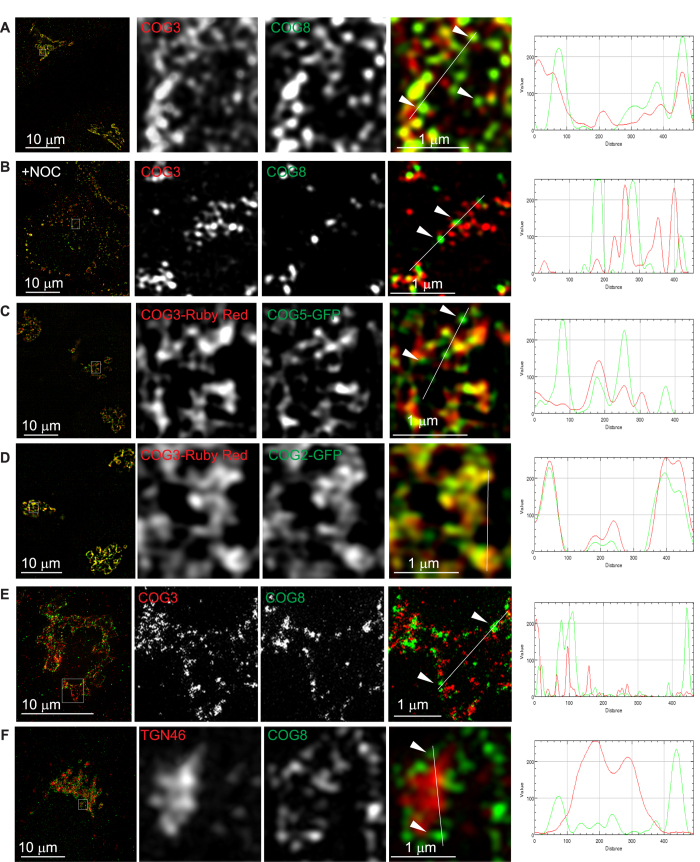
COG sub-complexes are spatially separated on Golgi membranes. HeLa cells stained for endogenous COG3 and COG8 (**A**,**B**,**E**), TGN46 and COG8 (**F**) or co-express siRNA resistant COG3-Ruby Red and COG5-GFP (**C**) or siRNA resistant COG3-Ruby Red and COG2-GFP (**D**) were imaged using the Zeiss ELYRA S1 (SR-SIM) (**A–D,F**) or the Leica TCS SP8 STED 3x microscope (**E**). In (**B**) cells were pre-treated with nocodazole (10 mM). Using a gene replacement strategy, cells were depleted of COG2 (**D**), COG3 (**C,D**) or COG5 (**C**) using siRNA transfection. 72 h after knockdown cells were transfected with siRNA resistant COG3-Ruby Red (**C,D**), COG5-GFP (**C**) or COG2-GFP (**D**), 24 h later cells were fixed and analyzed. Endogenous COG3, COG3-Ruby Red (labeled in red) as well as COG2-GFP were shown in elongated tubule like structures, likely indicating Golgi membranes. Endogenous COG8 and COG5-GFP (labeled in green) demonstrated a different pattern and appeared in punctuate vesicle-like structures (arrowheads). Line plots for overlap between red and green channels are shown measuring the relative value of signal intensity (y-axis) over the distance measured in pixels (x-axis). The intensity line profile of the Golgi region imaged by 3D**-**SIM was generated using ImageJ. Bar, 10 μm (inset 1 μm).

To overcome the resolution limits of standard confocal microscopy we employed two super-resolution techniques: structural illumination (3D SIM, Fig. 3A–D,F) and stimulated emission depletion (STED, Fig. 3E). In control HeLa cells, we observed partial co-localization of endogenous COG3 and COG8 signals in the Golgi region (Pearson coefficient (P) = 0.39 ± 0.09, n = 18), whereas the bulk of endogenous COG3 was detected on heterogeneous membrane structures, likely representing rims of Golgi cisternae. Endogenous COG8 was preferentially (~60%) associated with more uniform 50–120 nm structures, likely representing intra-Golgi transport vesicles or Golgi tubules (Fig. 3A, arrows). The estimated size of COG8 positive structures was close to the resolution limit of 3D SIM microscopy (~84 nm calculated in XY plane) therefore, we were unable to estimate the exact dimensions of these vesicle-like structures. Some of COG8 positive vesicle-like structures also demonstrated a residual amount of the COG3 signal possibly indicating a presence of the complete COG octamer connecting vesicle-like structures with the larger membranes. Importantly, the dramatic segregation of lobe A and lobe B signals on the Golgi was not an artifact of indirect fluorescent microscopy. Co-labelling cells with mouse and rabbit antibodies to the same COG3 subunit demonstrated much higher co-localization (Supplemental Figure 3A) (P = 0.68 ± 0.06, n = 10), while very low co-localization was observed between COG8 and trans-Golgi protein TGN46 (Fig. 3F) (P = 0.27 ± 0.09, n = 10) or between COG subunits and several other Golgi resident proteins (Supplementary Figure 3B). Separation of COG3 and COG8 signals was even more evident on Golgi mini-stacks in nocodazole-treated cells (Fig. 3B). Additionally, the observed COG subunit segregation was not an artifact of non-specific antibody binding, with similar separation of COG lobes demonstrated in cells created with a protein-replacement strategy that express COG3-Ruby Red and COG5-GFP instead of the endogenous subunits (Fig. 3C) (P = 0.72 ± 0.05, n = 13). Notably two lobe A subunits, COG3-Ruby Red and COG2-GFP, were much better co-localized on the Golgi membranes (Fig. 3D) (P = 0.84 ± 0.03, n = 10). Similarly, the STED approach revealed similar segregation of the endogenous lobe A (COG3) and lobe B (COG8) signals (Fig. 3E). In summary, both 3D SIM and STED super-resolution approaches revealed striking separation of lobe A and lobe B signals on Golgi membranes. These results strongly indicate that, in the steady state, a significant fraction of membrane-localized lobe B is separated from lobe A and preferentially associated with vesicle-like structures; while membrane-localized lobe A is localized on different membrane structures that may represent Golgi rims.

### COG lobe B sub-complex interacts with v-SNARE GS15 on Golgi vesicles

**Figure 4:**
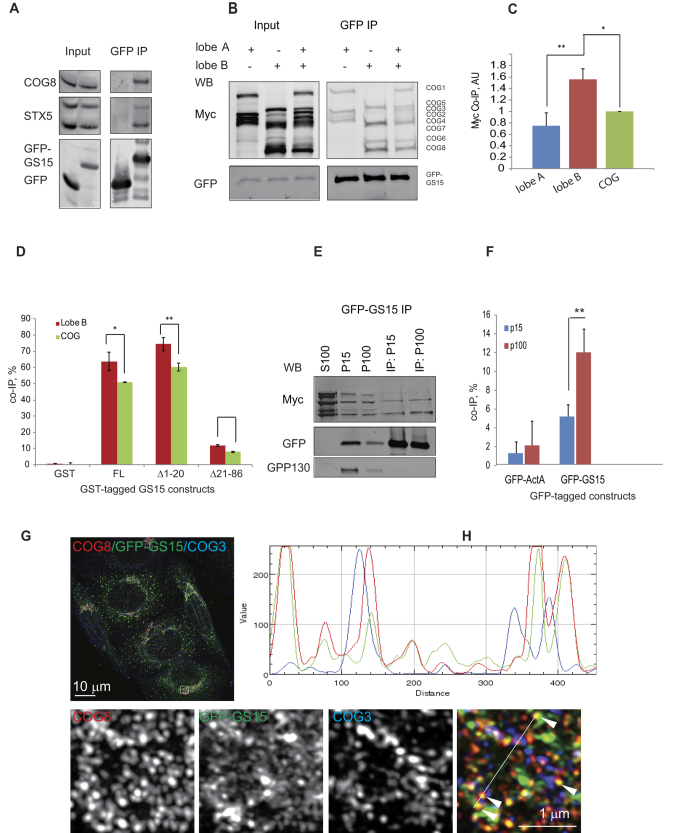
COG lobe B sub-complex preferentially interacts with GS15. (**A**) GFP-GS15 interacts with endogenous COG8. HEK293T cells were transiently transfected with GFP-GS15 or GFP then immunoprecipitated. IPs were probed by western blot with antibodies against COG8, STX5, and GFP. (**B**) GFP-GS15 preferentially interacts with lobe B *in vivo*. HEK293T cells were transiently transfected with COG-Myc multi-expression plasmids (lobe A expression, lobe B expression, or the whole COG complex). 24 h after COG-myc transfection cells were transfected with GFP-GS15. 24 h post transfection cellular lysates were immunoprecipitated. IPs were probed by western blot with antibodies against Myc and GFP. (**C**) Co-IP efficiency values were generated by calculating the relative density of the co-IP Myc signal relative to the density of the input Myc signal and the recovered GFP signal in four independent experiments. (**D**) COG-GS15 in vitro binding assay. Glutathione beads were incubated with recombinant GST, GST-tagged full-length (FL) GS15 cytoplasmic domain (aa 1–86), GS15 with deletion of N-terminal domain (Δ1–20), and deletion of SNARE domain (Δ21–86). Bound proteins were eluted from beads, and probed by western blot with Myc antibodies. IP efficiency values were generated by normalizing the density of the IP Myc signal to the density of the whole COG/Lobe B input Myc signal. (**E**) Lobe B interacts with GS15 on vesicle membranes. HEK293T cells were transiently transfected with lobe B and GFP-GS15. 48 h after transfection, cells were collected and fractionated into large membrane (P15), small membrane (P100), and cytosol (S100) by differential centrifugation. The membrane pellets were solubilized with Triton-X100 and then incubated with GBP-beads. IPs were probed by western blot with anti-Myc, anti-GFP, and anti-GPP130. (**F**) Co-IP efficiency values were calculated by dividing co-IP by input in four independent experiments. (**G**) HeLa cells stably expressing GFP-GS15 were stained for COG3 and COG8 and imaged using the Zeiss ELYRA S1. Line plots are shown measuring the relative value of signal intensity (y-axis) over the distance measured in pixels (x-axis). White arrows in the merged image indicate GS15 and COG8 co-localizing on vesicle-like structures. Bar, 10 μm (inset 1 μm).

Our data indicate that COG sub-complexes exist as separate entities on Golgi membranes/vesicle-like structures. This data is in good agreement with previous observations that t-SNARE STX5 and SM protein Sly1p interact with lobe A subunit COG4^18^,^19^ and that exogenously expressed COG sub-complexes preferentially interact with different vesicle-tethering factors^6^. The vesicle-like localization of the lobe B sub-complex suggests that lobe B subunits might interact directly or indirectly with vesicle-associated proteins involved in tethering/fusion, such as v-SNAREs. The major intra-Golgi v-SNARE is GS15/Bet1L^20^,^21^ which is the partner of STX5. Previous yeast two hybrid screens failed to identify COG as a partner for GS15^17^,^22^. However, GFP IP from cells that stably express GFP-GS15 revealed strong interaction with the endogenous lobe B subunit COG8 (Fig. 4A). Moreover, *in vivo* co-expression experiments showed that lobe B expressed in HEK293T cells preferentially binds to GFP-GS15 (Fig. 4B,C). This interaction is likely to be direct, as evidenced by purified GS15-GST very efficiently interacting with purified COG in the *in vitro* setting (Fig. 4D), with preferential binding to lobe B sub-complex compared to the full COG complex. Importantly, removal of the SNARE domain (aa 21–86) completely abolished this interaction, indicating that lobe B preferentially interacts with the SNARE domain of v-SNARE GS15. GS15 is present on both Golgi rims and Golgi-derived vesicles^20^; therefore we sought to determine the membrane compartment where lobe B interacts with GS15. Membranes from cells co-expressing GFP-GS15 and myc-tagged lobe B subunits were separated by differential centrifugation into P15 and P100 fractions (Fig. 4E). Golgi resident protein GPP130^23^ was found primarily associated with P15 fraction while GFP-GS15 was present in both P15 and P100 fractions (Fig. 4F, Supplemental Figure 4B), indicating that the P15 fraction is enriched in Golgi membranes while P100 contains Golgi-derived vesicles. Pull-down of GFP-associated proteins revealed that lobe B preferentially interacts with GS15 in the vesicle (P100) fraction (Fig. 4E,F). Vesicle-associated lobe B-GS15 interaction appeared to be strong (up to 12% pull-down) and highly specific for the v-SNARE GS15 with no significant interaction detected between lobe B and either Golgi tethering factor p115 (data not shown) or endosomal v-SNARE Vamp3 (Supplemental Figure 4B). In contrast to lobe B-GS15 vesicle association, lobe A subunit COG4 was shown to interact with Golgi t-SNARE STX5^17^ while COG2 directly binds to Golgi coiled-coil tethering factor p115^24^. P115 was originally found to be active in the *in vitro* intra-Golgi trafficking assay^25^ along with the COG complex^4^,^26^, indicating that these studies likely represent functional protein-protein interactions.

In order to complement the biochemical data, we employed superresolution microscopy (Fig. 4G and Supplemental Figure 4C). In HeLa cells stably expressing GFP-GS15, a sub-population of lobe B subunit COG8 (P = 0.59 ± 0.09, n = 15), but not lobe A subunit COG3 (P = 0.29 ± 0.04, n = 15), was detected on GS15-positive vesicle-like structures (white arrowheads on the overlay insert). In summary, these results indicate that lobe B of the COG complex preferably interacts with v-SNARE GS15, and furthermore that this interaction is likely occurring on vesicle membranes.

### Transient COG1-COG8 interaction is essential for the COG complex function

**Figure 5:**
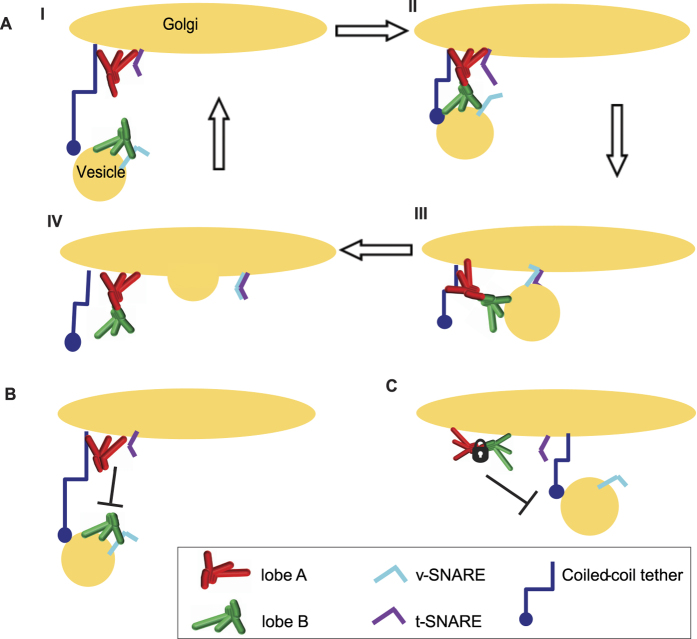
Hypothetical model of lobe A-lobe B interaction mediated vesicle tethering. Hypothetical model of how lobe A and lobe B interaction mediates the tethering of an incoming retrograde vesicle. (**A**) Incoming vesicle containing a v-SNARE (example GS15) and lobe B sub-complex, will begin its tethering via interaction with a coiled-coil tether (example P115) from a further distance (**A-I**). Coiled-coil tether vesicle sequestration pulls the vesicle into close enough proximity to the Golgi membrane that lobe A and lobe B on opposing membranes will interact (**A-II**). Interaction between lobe A and lobe B (via COG1-COG8 direct protein-protein interaction) organizes the vesicle for proper alignment of the v-SNARE to the pre-assembled t-SNARE complex (example STX5/GS27/GS28/GS15 containing SNARE complex) (**A-III**). Aligned SNAREs then allow for vesicle docking and SNARE-pin formation to drive vesicle fusion (**A-IV**). If lobe A-lobe B interaction is required for COG mediated vesicle tethering, then defects in lobe A-lobe B interaction (**B**), or stabilization of octameric COG (**C**) would block the productive tether of incoming vesicle and prevent its fusion with the Golgi.

Our biochemical and microscopy data suggest that COG sub-complexes are largely separated on different membranes, with lobe A readily present on large membrane structures (likely Golgi cisternal rims) and lobe B enriched on GS15-containing vesicle-like structures. Based on this separation, we propose a model of COG complex mediated vesicle tethering that utilizes a transient interaction between lobe A and lobe B sub-complexes on opposing membranes to bridge a vesicle to an acceptor Golgi membrane (Fig. 5A). The model predicts that inhibition of efficient COG complex assembly or disassembly will interfere with its function. To test this model, we designed a set of experiments to either restrict COG complex assembly by destroying the lobe A-lobe B interaction bridge (Fig. 5B) or to prevent disassembly by locking the complex in its octameric arrangement (Fig. 5C). We hypothesize that in both cases the COG complex would be incapable in organizing/tethering an incoming vesicle^27^, and thus would interfere with intra-Golgi trafficking, detected by the mis-glycosylation of the cells total glycoconjugates^9^,^28^.

**Figure 6:**
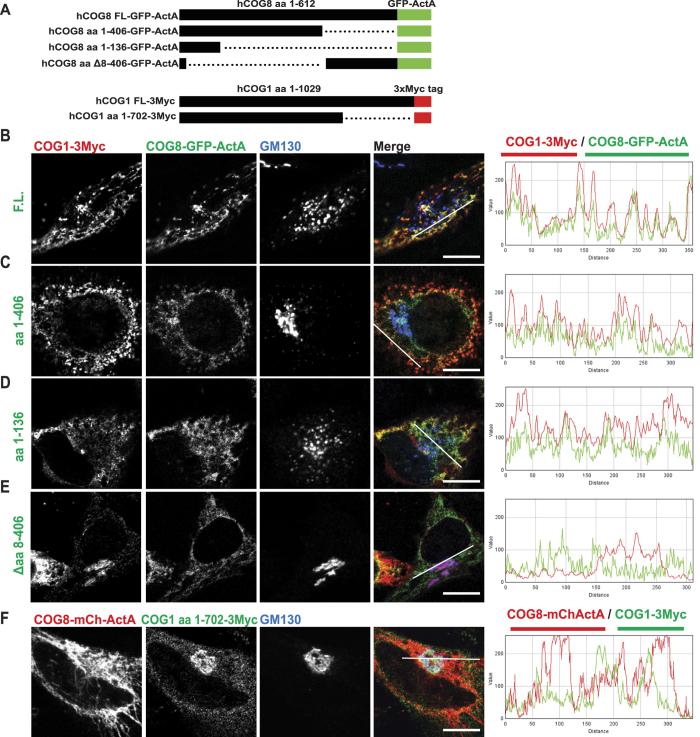
The C terminus of COG1 interacts with the N terminus of COG8. (**A**) Schematic of the constructs used in the mitochondrial relocalization assay: Full length (FL) COG8, COG8 aa 1–406, COG8 aa 1–136, or COG8 Δaa8–406 tagged with GFP-ActA. COG1 full length (FL) or 1–702 tagged with 3Myc. HeLa cells were transiently transfected with plasmids as follows: (**B**) hCOG1-Myc and hCOG8-GFP-ActA, (**C**) hCOG1-Myc and hCOG8(1–406)-GFP-ActA, (**D**) hCOG1-Myc and hCOG8(1–136)-GFP-ActA, (**E**) hCOG1-Myc and hCOG8(Δ8–406)-GFP-ActA, (**F**) hCOG8-mChActA and hCOG1(1–702). 24 h after transfection cells were fixed, and then stained with antibodies to Myc and GM130 and analyzed by confocal microscopy. Line plots for overlap between red and green channels are shown. Size bar, 10 μm.

To test this hypothesis, we first identified the protein domains that are responsible for the bridging of COG sub-complexes. Previous *in vitro* and *in vivo* analysis suggested that the major interaction between lobe A and lobe B occurs via COG1-COG8 direct protein-protein binding^8^,^29^. To test the COG1-COG8 interaction in live cells we employed a mitochondria relocalization assay^22^,^30^. Several different deletions of mitochondria targeted COG8-GFP-ActA (Supplemental Figure 6A) were co-expressed with the full length myc-tagged COG1 in HeLa cells (Fig. 6A–E). We detected that COG1 was re-directed and co-localized with full-length COG8-GFP-ActA on the mitochondria (Fig. 6B) confirming the assay. Similar co-localization was observed for COG8 mutants that contain the first 406, or even 136, amino acids of COG8 (Fig. 6C,D) indicating that the N-terminal part of COG8 is sufficient for COG1-COG8 interaction. In agreement with this, depletion of the N-terminal portion of COG8 (Fig. 6E) completely abolished COG1-COG8 co-localization on the mitochondria, indicating that the first 406 amino acids of COG8 are essential for the COG1-COG8 interaction. In agreement with this proposal COG8 lacking amino acids 8–406 failed to localize to the Golgi while a COG8 mutant lacking the C-terminal domain (aa 537–614)^29^ was predominantly found on the Golgi membranes (Supplemental Figure. 6C). While the full length COG1 was faithfully recruited by the mitochondrial-targeted COG8-mCherry-ActA (Supplemental Figure 6B), depletion of the C-terminal 227 amino acids of COG1 similarly abolished COG1-COG8 co-localization (Fig. 6F) supporting the model in which the COG1 C-terminus is interacting with the N-terminal portion of COG8. We verified that the COG1-COG8 interaction was the sole bridging mechanism for lobe A and lobe B sub-complexes by co-expression of myc-tagged COG subunits with COG3-GFP in ΔCOG1 cells. GFP pull-down in the absence of COG1 did not co-IP lobe B subunits, confirming that COG1 is the essential bridge for lobe A-lobe B co-recovery (Supplemental Figure 5).

**Figure 7:**
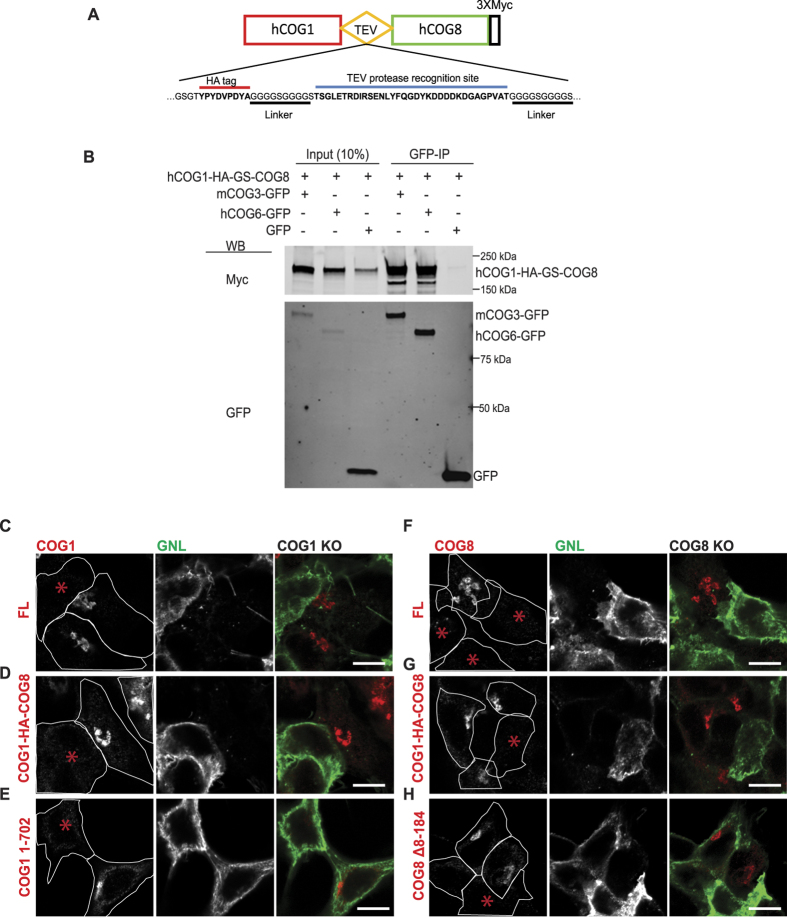
COG1-COG8 hybrid is incorporated into COG complex and properly localized to Golgi membranes. (**A**) Schematic of the COG1-HA-GS-COG8 hybrid construct with flexible linker and TEV protease recognition site. (**B**) HEK 293 T cells were co-transfected with COG1-HA-GS-COG8 hybrid and either mCOG3-GFP, hCOG6-GFP, or GFP as a negative control. Cell lysates prepared 24 h after transfections were incubated with GBP-beads. IPs, along with 10% of total input, were separated on an SDS–PAGE gel, and blotted with anti-Myc (upper panel) and anti-GFP (lower panel) antibodies. ΔCOG1 cells were transfected with plasmids encoding either COG1-3Myc (**C**), COG1-HA-GS-COG8 hybrid (**D**) or COG1 1-702-3Myc (**E**). ΔCOG8 cells were transfected with plasmids encoding either COG8-3Myc (**F**), COG1-HA-GS-COG8 hybrid (**G**), or COG8 Δ8-184-3Myc (**H**). 72 hours after transfection cells were fixed and surface labeled with GNL-647 and stained with anti-Myc antibodies for confocal microscopy analysis. Cell outlines overlaid with the Myc channel. COG1-HA-GS-COG8 hybrid is capable of rescuing the glycosylation defect in both ΔCOG1 and ΔCOG8 cells. COG1 or COG8 lacking the interaction site for their respective partner will not rescue the COG subunit KO glycosylation defect phenotype. Size bar, 10 μm. * Denotes a non-transfected/un-rescued KO cell.

In order to prevent the dissociation of lobe A and lobe B sub-complexes, we designed a hybrid construct permanently connecting the C-terminus of COG1 to the N-terminus of COG8 via a HA-tag containing a flexible GS linker and TEV protease recognition site (Fig. 7A). Upon co-expression with GFP-tagged COG3 or COG6 in HEK293T cells the COG1-HA-GS-COG8 hybrid was efficiently co-precipitated with both lobe A and lobe B subunits, indicating that the hybrid molecule folded properly, is capable of essential interactions with its respective COG partners and therefore properly incorporated into the COG complex (Fig. 7B).

Next we tested the ability of the hybrid protein, as well as C-terminally deleted COG1 and N-terminally deleted COG8, to rescue glycosylation defects associated with the absence of either COG1 or COG8 proteins. ΔCOG1 or ΔCOG8 HEK293T cells lines were generated by CRISPR technology (see Materials and Methods) and were characterized by partial genome sequencing (Supplemental Figure 7A) and western blotting^14^. We and others have previously shown that COG complex subunit knockdowns (KD) in HeLa and HEK293T cells cause altered binding of several lectins due to impaired glycosylation of plasma membrane glycoconjugates while in the Golgi^9^,^10^,^15^,^28^,^31^. We thus used this to characterize COG specific glycosylation defects in COG KO cells. Indeed, mutant HEK293T cells did not show any growth abnormality but, unlike control cells, their plasma membrane was uniformly stained with mannose-specific *Galanthus nivalis* lectin conjugated with Alexa 647 (GNL-647), indicating a COG-dependent glycosylation defect^9^,^28^ (Supplemental Figure 7B). GNL binds to terminal α1-3 linked mannose residues^32^ in all tested COG KD^9^ and KO^33^ cells making it a helpful probe for immature glycans. The increased amount of plasma membrane glycoconjugates with terminal α1-3 linked mannose residues indicates altered activities in *cis*-Golgi Mannosidase I enzymes as well as in *cis*/medial-Golgi localized Mannosidase II (MAN2A) and/or GlcNAc-T1 (MGAT1) transferase that were shown previously to be COG complex dependent^9^. Importantly, both the lobe A deficient ΔCOG1 cell line and lobe B deficient ΔCOG8 cell line demonstrated similar robust glycosylation defects indicating that both COG sub-complexes are essential for proper protein glycosylation in the Golgi. As expected, glycosylation defects in both ΔCOG1 and ΔCOG8 cell lines were rescued by the expression of myc-tagged full-length COG1 or COG8 molecules (Fig. 7C,F). Similar results were obtained by expressing the COG1-HA-GS-COG8 hybrid (Fig. 7D,G), indicating that the hybrid molecule can functionally substitute the individual deleted proteins, confirming proper folding of both parts of the COG1-COG8 chimera. In both cell lines COG1-HA-GS-COG8 hybrid was properly localized to the Golgi area. Although the COG1-COG8 hybrid still shows some interaction with GFP-GS15 (Supplemental Figure 7C), the interaction with this v-SNARE was severely reduced as compare to COG8-myc (~0.3% for the hybrid compared to 5–8% for COG8-myc in Fig. 4) indicating possible functional defects of COG in a locked lobe A-lobe B configuration. Strikingly, expression of C-terminally deleted COG1 (Fig. 7E) or N-terminally deleted COG8 (Fig. 7H) did not rescue the glycosylation defect, supporting our hypothesis that bridging COG sub-complexes is essential for COG function. Importantly, in both cases, truncated molecules were properly localized to the Golgi region, indicating sufficient expression and unhampered interaction with other Golgi-localized trafficking machinery. Interestingly, expression of C-terminally truncated COG8 was able to significantly reduce GNL binding indicating that this part of COG8 is dispensable for the activity/localization of medial-Golgi enzymes (Supplemental Figure 7D).

**Figure 8:**
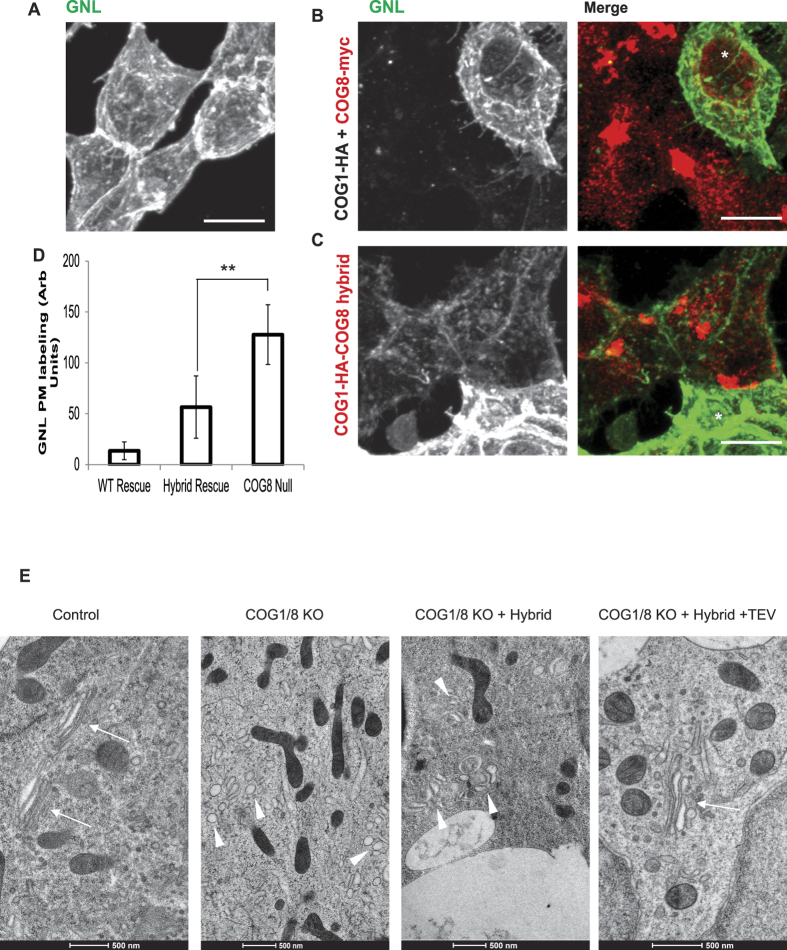
COG complex disassembly is critical for its role in Golgi glycosylation and stability. ΔCOG8 cells (**A**) were transiently transfected with siRNA against COG1 (**B,C**) and then co-transfected with plasmids encoding COG1-3Myc and COG8-3Myc (**B**) or COG1-HA-GS-COG8 hybrid (**C**). 72 hours after transfection cells were fixed, surface labeled with GNL-647, and stained with anti-Myc antibodies for confocal microscopy analysis. The recovery of the ΔCOG mediated glycosylation defect was quantified by line scan analysis using Fiji plot profile and the averages are shown in the graph (**D**). 12–14 cells were analyzed with three measurements per cell. Error bars denote standard deviation. * Denotes control un-transfected cell. (**E**) TEM images of the Golgi region. Control HEK293T cells (control), ΔCOG1/ΔCOG8 (COG1/8 KO) or ΔCOG1/ΔCOG8 cells transfected with the COG1-HA-GS-COG8 hybrid alone (COG1/8 KO + hybrid), or transfected with COG1-HA-GS COG8 Hybrid and hTEV plasmid (COG1/8 KO + Hybrid + TEV) were grown on sapphire discs for 3 days, subjected to the HPF-FS procedure and embedded in resin. Thin-sections were cut at 50 nm and images at 11,500x. Golgi stacks we observed in 80% of control cells (n = 10) and in 37% of ΔCOG1/Δ COG8 + Hybrid + TEV cells (n = 8) but not in ΔCOG1/Δ COG8 or ΔCOG1/Δ COG8 + hybrid cells (n = 10). Note Golgi stacks (arrows) and Golgi-like dilated membranes (arrowheads).

To test if the COG1-HA-GS-COG8 hybrid can simultaneously replace both COG1 and COG8 subunits, ΔCOG8 cells were treated with siRNA to COG1, and then transfected with tagged COG subunits (Fig. 8A–C). Again, ΔCOG8 siCOG1 cells were intensely stained with GNL-647 indicating a glycosylation defect and confirming the depletion of the subunits (Fig. 8A). The GNL-binding defect was completely corrected by 5 days co-expression of independent COG1 and COG8 (Fig. 8B), but only partially corrected by expression of the COG1-HA-GS-COG8 hybrid (Fig. 8C). Quantification of GNL-647 staining (Fig. 8D) revealed that cells expressing COG1-HA-GS-COG8 hybrid had significantly more GNL plasma membrane binding compared to the unlinked rescuers. These results indicate that permanent linkage between lobe A and lobe B COG sub-complexes is detrimental for COG complex function in directing intra-Golgi trafficking of glycosylation machinery.

WB analysis revealed that the COG1-HA-GS-COG8 hybrid was stable in cells 48 h after transfection, but partially proteolytically cleaved/degraded after 4 days (Supplemental Figure 8A), releasing small amounts of full-length COG8 which may contribute to a partial rescue of the glycosylation defect observed in COG deficient cells expressing the COG1-HA-GS-COG8 hybrid. This undesired cleavage may correspond to a cryptic caspase cleavage site recently found in the HA epitope^34^. Our COG1-HA-GS-COG8 hybrid construct contains a TEV protease cleavage site in the middle of a flexible linker and its cleavage was dramatically accelerated in cells co-transfected with a construct expressing TEV protease (Supplemental Figures 7E and 8A). We utilized this property of the COG1-COG8 hybrid to compare the rescue of COG-related glycosylation defects in cells transfected for a short time either with the COG1-COG8 hybrid construct alone, or co-transfected with TEV protease in COG1/COG8 double KO HEK293T cells (Supplemental Figure 8B). FACS analysis of GNL-647 treated cells revealed that at 48 h the COG1-COG8 hybrid failed to rescue COG1/COG8 null-mediated glycosylation defects (Supplemental Figure 8B, Hybrid only population, green line) while the unlinked COG1 and COG8 released by TEV protease were capable of partially alleviating GNL-binding of plasma membrane proteins (Supplemental Figure 8B, Hybrid + TEV population, red line). Partial rescue of the glycosylation defect observed in ΔCOG1/COG8 cells co-transfected with COG1-HA-GS-COG8 hybrid and TEV protease plasmids is likely due to a short expression time and a slow renewal rate of plasma membrane glycoconjugates.

To further test the functionality of the COG1-HA-GS-COG8 hybrid we employed transmission electron microscopy (TEM) of high pressure frozen samples (Fig. 8E). In control HEK293T cells Golgi membranes appeared as a tight perinuclear ribbon-like structure (Fig. 8E, control, arrows) while in double COG1/COG8 KO cells only multiple round vesicle-like structures were found in the perinuclear region (Fig. 8E, COG1/8 KO, arrowheads). This phenotype was not significantly improved after 3 days expression of COG1-HA-GS-COG8 hybrid (Fig. 8E, ΔCOG1/8 + Hybrid). Importantly co-expression of COG1-HA-GS-COG8 hybrid and TEV protease restored Golgi stacks morphology (Fig. 8E, ΔCOG1/8 + Hybrid + TEV, arrow).

In order to circumvent the cryptic HA cleavage site, we substituted the HA tag with GFP, allowing to sort for the population of cells expressing the hybrid. Consistent with the initial construct, the COG1-GFP-GS-COG8 construct was not functional in rescuing COG1/COG8 double KO cells while the unlinked COG1 and COG8 were capable of significantly decreasing GNL-647 (Supplemental Figure 8C). Based on these results we conclude that the locked octameric complex is not sufficient to rescue both ΔCOG1 and ΔCOG8 cells.

In summary, these results are supportive of our hypothesis that lobe A-lobe B sub-complexes have a transient interaction, and that both assembly and disassembly steps are required for proper COG complex function.

## Discussion

Multi-subunit tethering complexes participate at every step of intracellular membrane trafficking but the exact molecular dynamic of these complexes during the tethering process and the mechanism of their tethering action is largely unknown. While in live cells these complexes are present in both soluble and membrane-bound forms^35^,^36^,^37^,^38^ it was predicted that the membrane-bound form is the active one that tethers transport intermediates. For the soluble COG complex both subunit composition and intra-complex interactions were studied previously^3^,^4^,^5^,^8^,^16^ while the composition of the membrane-bound form(s) was not thoroughly investigated.

In this study, we have discovered that membrane-bound COG complex lobe A and lobe B exist as independent sub-complexes in addition to their well characterized configuration in the octameric complex. Multiple lines of evidence indicated that membrane-bound lobe A subunit COG3 is significantly segregated from lobe B subunits COG5, COG6 and COG8 while remaining tightly bound/co-localized to its partner proteins COG2 and COG4. This steady-state arrangement of membrane-bound COG subunits could be explained in three different models. In the first model, both complex and sub-complexes are permanently present on different Golgi sub-compartments to tether different populations of intra-Golgi vesicles. Indeed a recent study described differences in stability of Golgi glycosyltransferases in cells depleted for either COG3 (lobe A) or COG7 (lobe B) subunits^39^. However, a subsequent study that comprehensively compared cells depleted for either lobe A or lobe B subunits did not reveal any major sub-complex-specific differences in either stability of Golgi enzymes or in protein glycosylation^9^. Moreover, HEK293T cells completely depleted for either lobe A subunit COG1 or lobe B subunit COG8 demonstrated very similar glycosylation defects^14^ . This model also does not explain the existence of the large soluble pool of the predominantly octameric COG complex.

In the second model, the complete COG complex plays an active role in vesicle tethering reactions and becomes inactivated by a separation to smaller sub-complexes. This model does not agree with our finding that lobe B subunits are observed on vesicle-like structures, likely to be the previously observed COG complex dependent (CCD) vesicles^40^.

In the third model, the COG complex mediates vesicle tethering via a transient interaction between lobe A and lobe B sub-complexes on opposing membranes to bridge a vesicle to the acceptor Golgi membrane (Fig. 5A). In this model, membrane-bound sub-complexes are active components in the vesicle tethering/proofreading reaction while the octameric complex serves as an inert reservoir, waiting to be recycled back to the active units (sub-complexes). We favor this third model and believe that our data support this scenario. Indeed, both microscopy and biochemical data indicate that a fraction of lobe B travels on smaller vesicle-like structures where it specifically interacts with vesicle-associated SNARE GS15. In contrast, lobe A is preferentially located on different membrane structures (likely to represent the rims of Golgi cisternae) where it interacts with coiled-coil tether p115^24^, t-SNARE STX5^17^ and SM protein Sly1^19^. The preference in the partner interactions is indicative of the separation of the sub-complexes. Similarly, we have previously demonstrated that there is a difference in the kinetic mobility of lobe A vs lobe B subunits^6^. Fluorescence recovery after photo-bleaching (FRAP) of Golgi localized COG3 had a slower rate than COG8, which we interpreted as a tighter association of lobe A to Golgi membranes than lobe B. We can now extend this result to propose that a subpopulation of lobe B is present on vesicle-like membranes, and that fusion of these lobe B-containing small carriers with Golgi membranes is responsible for the faster COG8 recovery time. While we favor this model as the main mode of the COG complex function in trafficking of Golgi enzymes, some of our data can be interpreted in favor of the alternative model(s) in which both the COG complex and sub-complexes function on different Golgi sub-compartments to tether different populations of intra-Golgi vesicles^10^,^39^,^41^. It is possible that the COG1-COG8 hybrids used in our studies are not flexible enough to recapitulate a natural COG1-COG8 complex and that tethering the ends of these proteins together could affect conformational changes of the octamer. Further in vitro experiments will answer these challenges and provide a robust mechanistic model of COG complex function.

There is evidence of another CATCHR complex functioning in a similar mechanism to our proposed COG model. The mammalian Exocyst complex has similarly been shown to be divided into sub-complexes on opposing membranes; Sec15, Exo84, and Sec10 on an incoming vesicle membrane and Sec3, Sec5, Sec6, Sec8, and Exo70 on the plasma membrane^42^. The authors of this study proposed that the direct interaction between sub-complexes bridged the vesicle to the membrane. While this is an intriguing model within the field, what has been lacking is the isolation of the exocyst sub-complexes^43^. Recently however, the yeast Exocyst was shown to be an assembly of two four-subunit complexes^44^, similar to our model of COG tetrameric sub-complexes.

Recent data from the Novick lab indicate that the v-SNARE Snc2 specifically recruits the Exocyst (or its sub-complex) to secretory vesicles^45^, providing another similarity to the COG complex mode of action. Our data showed that COG interacts with the major Golgi v-SNARE GS15, and that this interaction is robust for lobe B in a vesicular fraction. The exact mode of v-SNARE-tether interaction appears to be similar for COG and Exocyst complexes: Exocyst is binding to the SNARE domain of Snc2^45^, while COG lobe B requires the SNARE domain of GS15 for efficient binding (potentially regulating/proofreading its incorporation into a functional SNARE complex). It is important to note here that both GS15 localization and stability are strongly dependent on COG function^40^,^46^, indicating that direct GS15-lobeB interaction is essential for Golgi v-SNARE function. The exact mechanism of COG-GS15 interaction requires further investigation.

In the proposed model, the bridging of lobe A and lobe B together provides the physical tethering property of COG. As a result, the octameric complex is probably a by-product of membrane fusion and would therefore be an inert conformation. If this prediction is correct, then the octameric complex must be disassembled for subsequent rounds of vesicle tethering. Our data indicate that the complex is disassembling into sub-complexes, not individual subunits. Thus, there must be a step of the COG complex cycle that disrupts the COG1-COG8 interaction and separates the complex. One possibility here is the presence of a yet unidentified Golgi-associated COG “splittase” that separates COG lobes on the membrane. Another possibility is that the multipronged interaction of lobe A with its Golgi partners (STX5, SLY1, P115, Rab30)^6^,^17^,^19^,^24^,^47^ and simultaneous interaction of lobe B with its vesicular partners (GS15, COPI, Rab2)^6^,^47^ during the budding of recycling vesicles is sufficient to change the COG conformation to a state that favors separate stable sub-complexes. As a result of the separation an activated lobe B would end up on recycling COPI-vesicles while lobe A would stay at the Golgi “docking stations”, awaiting the next round of approaching vesicles. Identifying the exact location of COG complex segregation and the nature of proteins regulating COG disassembly will be an on-going topic of research.

## Materials and Methods

### Reagents and Antibodies

*Galanthus nivalus* lectin (GNL) (20 μg/ml, Vector) was labeled with an Alexa-647 protein labeling kit from Life Technologies.

Antibodies used for immunofluorescence (IF) microscopy, western blotting (WB) and immunoprecipitation (IP) were purchased through commercial sources, gifts from generous individual investigators, or generated by us via affinity purification. Antibodies were as follows: mouse monoclonal antibodies: β-Actin (Sigma), COG3 (this lab), GFP (BioLegend), GM130 (BD Biosciences), GAPDH (Santa Cruz), Vti1a (BD Biosciences); rabbit polyclonal antibodies: Myc (Bethyl), COG3 (this lab), COG4 (this lab), COG5 (Daniel Ungar, University of York), COG6 (this lab), COG8 (this lab), COG8 (Sigma), GPP130 (Alexis), STX5 (this lab).

Secondary IRDye 680 goat anti-rabbit, IRDye 800 donkey anti-rabbit, and IRDye 700 goat anti-mouse for WB were obtained from LI-COR Biosciences. Donkey anti-mouse DyLight 647, donkey anti-mouse Cy3, and donkey anti-rabbit Cy3 for IF were purchased from Jackson Immuno Research. Goat anti-mouse Alexa 488 and goat anti-rabbit Alexa 488 for IF were purchased from Rockland.

### Cell culture

HeLa and HEK293T cells (ATCC) were cultured in DMEM/F-12 medium (Thermo Scientific) supplemented with 15 mM HEPES, 2.5 mM L-glutamine and 10% FBS (Atlas Biologicals) at 37 °C and 5% CO_2_ in a 90% humidified incubator. YFP-COG3/CFP-COG6 stable cells were generated by transfection of CFP-COG6 plasmids into HeLa cells stably expressing YFP-COG3^37^ followed by selection with Zeocin and single colony isolation.

HEK293T COG1 and COG8 stable knockouts were generated using CRISPR^48^,^49^,^50^. gRNA sequences were provided by Horizon Discovery, COG8 Guide ID:123842 GGTGGAGGATGAAGGGCTCC, COG1 Guide ID: 129696 TTTCGAGACGCATGGAGCGG. HEK293T cells were transfected with plasmid containing gRNA with Cas9-dasherGFP (Horizon Discovery). Eight days after transfection, COG depleted cells were identified by increased cell surface binding of GNL-647 and single-cell sorted based on high 647 fluorescence intensity using the FACSARIA (BD Biosciences). Single cell colonies were then expanded. Cells were verified as nulls by WB and sequencing. To create the COG1/COG8 double KO cell line the COG1 KO cells obtained in the first round of selection were transfected again with COG8 gRNA/Cas9 construct, single sorted and verified by WB and sequencing.

### Size exclusion chromatography

Size-exclusion chromatography was performed using Superose 6 10/300 GL column with the AKTA purifier FPLC (GE Healthcare). The column was equilibrated in column buffer (125 mM Potassium Acetate, 25 mM HEPES, pH 7.2). Ferritin from equine spleen (Sigma, Type I, saline solution), alcohol dehydrogenase (Worthington Biochemical Corporation), and bovine serum albumin (RPI) diluted with column buffer with 0.1% Tween 20 to 10 mg/mL were used for column calibration. The void volume (Vo) at ~7.5 mL was used with the elution volume (Ve) to generate a protein standard curve to calculate the size of eluted proteins.


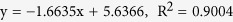


Sample preparation: HeLa cells were washed with DPBS and collected by scraping in column buffer with Halt protease inhibitor cocktail (Thermo Scientific) and 1 mM PMSF. Collected cells were lysed by vortexing with 0.5 mm glass beads (BioSpec) on ice. Total cell lysates were centrifuged at 660xg for 5 min at 4 °C to isolate the post-nuclear supernatant (PNS). PNS was separated into supernatant (cytosol) and pellet (membrane) fractions by ultracentrifugation at 100,000xg for 1 hour at 4 °C in a Beckman Optima ultracentrifuge using TLA 55 rotor. Supernatant (cytosol fraction) was concentrated with a NanoSep 30 K centrifugal device for 20 min at 20,000 × g at 4 °C (~10 fold concentration). Membrane pellet (membrane fraction) was suspended in 1%Triton X-100 in DPBS with protease inhibitors, spun down at 20,000xg at 4 °C, and solubilized membranes collected. Triton X-100 (final concentration 1%) was added to cytosol fraction as well. Cytosol fraction and Triton X-100 soluble membrane fraction were loaded on column equilibrated with column buffer plus 0.1% Tween 20 at 0.4 mL/min, 0.5 mL fractions were collected after 0.35 CV elution. Fractions were concentrated by TCA precipitation (trypsin inhibitor added to final concentration of 0.01 μg/μL, 5 μL 0.02% sodium deoxycholate (Sigma) added and samples incubated for 15 min on ice. 168.5 μL of 24% TCA added and samples incubated on ice 1 hour. Precipitated proteins were collected by centrifugation at 20,000x*g* for 20 min at 4 °C and resuspended in 45 μl sample buffer.

### Native IP of endogenous COG subunits from intracellular membranes

HeLa cells were collected on ice in DPBS containing Halt protease inhibitor cocktail (Thermo Scientific), and lysed by vortexing with 0.5 mm glass beads (BioSpec). Total cell lysates were centrifuged at 660 × g for 5 min at 4 °C to isolate the post-nuclear supernatant (PNS). PNS was separated into supernatant (cytosol) and pellet (membrane) fractions by ultracentrifugation at 100,000 × g for 1 hour at 4 °C in a Beckman Optima ultracentrifuge using TLA 55 rotor. Membrane pellet (P100) was then resuspended in 1 ml of DPBS containing 1% Triton X-100 and Halt protease inhibitor cocktail, and incubated for 30 min on ice. Triton X-100 (final concentration 1%) was added to cytosol fraction as well. 30 μl of protein A-Sepharose CL-4B beads was added to both supernatant and pellet fractions and incubated with gentle mixing by inversion for 1 h on ice and finally clarified by centrifugation at 20,000 × *g* for 10 min to produce S20 (for cytosolic proteins) and P20 (for detergent-soluble membrane proteins) fractions. S20 and P20 were mixed with 3 μg of affinity purified anti-hCOG3, or anti-hCOG6 antibodies and incubated overnight on ice. Samples were clarified by centrifugation as described above. 60 μl of Protein G-Agarose beads slurry (50%) was added to each tube and incubated for 2 h with gentle inversion on the rotator in a cold room. Beads were then washed four times with TBST. Samples were eluted in 2X sample buffer, and heated for 5 min at 95 °C to elute the bound proteins.

### siRNA-induced knockdown

Transfection was performed using RNAiMAX siRNA Transfection Reagent (Life Technologies), following a protocol recommended by Life Technologies and cells were analyzed 72–96 hours after transfection. (siRNA Human COG1-4: GUAGCGGCCUCUCCAUGAA).

### Plasmid preparation and transfection

Mammalian expression constructs were generated using standard molecular biology techniques or obtained as generous gifts. See Table 1 for complete list of plasmids. Plasmids were isolated from bacterial cells using the QIAprep Spin Miniprep or Midiprep Kits (Qiagen). Plasmid transfections were performed using Lipofectamine 2000 (Life Technologies).

### Fractionation of mechanically disrupted cells

HEK293T cells were collected by gentle resuspension and pelleted by centrifugation at 500xg for 2 min. Cell pellet was resuspended in cold homogenization buffer (25 mM HEPES-KOH pH 7.2, 150 mM KCl, 2 mM EDTA, 5 μL/mL Halt protease inhibitor cocktail (Thermo Scientific), and 1 mM PMSF). Cells were lysed by passage through a 27 G needle 30 times, or until 90% of cells were broken. Cell lysate was centrifuged at differential speeds as follows: the whole cell lysate was centrifuged 500 × g for 5 min at 4 °C to remove unbroken cells and nuclei. Remaining supernatant (S0.5) was centrifuged at 15,000 × g for 10 min at 4 °C to pellet large membranes including Golgi (P15). S15 was ultracentrifuged at 100,000 × g for 1 hour at 4 °C in a Beckman Optima ultracentrifuge using TLA 55 rotor to pellet remaining membranes including vesicles. Recovered cell pellets were washed with homogenization buffer and resuspended in 0.5 mL of lysis buffer (50 mM Tris, 150 mM NaCl, pH 7.4) containing 1% Triton and 5 μL/mL Halt protease inhibitor cocktail (Thermo Scientific), and 1 mM PMSF and incubated on ice for 30 min. Cell lysates were cleared by centrifugation at 20,000xg and recovered supernatants used for subsequent analysis.

### Immunofluorescence microscopy

Cells were grown on 12 mm glass coverslips (#1, 0.17 mm thickness) one day before transfection. After transfection cells were fixed and stained as described previously^9^. In short, cells were fixed in 4% paraformaldehyde (16% stock solution; Electron Microscopy Sciences). Cells were then treated with 0.1% Triton X-100 for one minute. After that the cells were treated with 50 mM ammonium chloride for 5 min. Cells were washed with DPBS and blocked twice for 10 min with 1% BSA, 0.1% saponin in DPBS. Cells then were incubated for 1 hour with primary antibody diluted in antibody buffer (1% cold fish gelatin, 0.1% saponin in DPBS) at room temperature. Cells were washed four times with DPBS and incubated for 30 min with fluorescently tagged secondary antibody in antibody buffer at room temperature. After that, coverslips were washed four times with DPBS, rinsed with ddH_2_O, and mounted on glass microscope slides using Prolong^®^ Gold antifade reagent (Life Technologies). Cells were imaged with the 63X oil 1.4 numerical aperture (NA) objective of a LSM510 Zeiss Laser inverted microscope outfitted with confocal optics and four lines of excitation lasers (405, 488, 561, and 642 nm) under the control of Zen 2009 software (Zeiss). The pixel size was 93 nm. The “RGB profiler” plug-in of ImageJ (http://rsbweb.nih.gov/ij) was used to generate line plots for individual channels. The “Coloc_2” plug-in was utilized to calculate Pearson coefficient for co-localization. At least three independent experiments were performed to calculate both mean and standard deviation values.

### Live Cell Microscopy

HeLa cells that stably express YFP-COG3 and CFP-COG6 were grown on glass-bottom culture plates. Live cells were imaged using a laser-scanning confocal microscope (LSM 510 Zeiss Laser inverted microscope outfitted with confocal optics) equipped with an environmental control system (Live cell system, BioVision Technologies) set to 37 °C and 5% CO_2_. Cells were cultured in phenol red–free DMEM/F12 (Sigma-Aldrich) with 1% FBS. Brefeldin A (BFA) (final concentration of 0.25 μg/mL) in 10% FBS in DMEM/F-12 medium without antibiotics was added to cells in glass-bottom culture plates. Cells were imaged after 15 min incubation.

### Super-resolution Microscopy

Cells grown on coverslips were fixed and labeled with affinity-purified antibodies to COG3 and COG8 as detailed above. Cells co-expressing fluorescent protein tagged COG subunits were initially transfected with siRNAs to corresponding subunit. 48 h later cells were co-transfected with DNA plasmids expressing siRNA-resistant tagged COG subunits. 24 h later cells were fixed and analyzed. 3D-SIM samples were labeled with mouse monoclonal anti-COG3 and rabbit polyclonal affinity purified anti-COG8 primary and anti-mouse Cy3b and anti-rabbit-Alexa488 secondary antibodies. STED samples were labeled with mouse monoclonal anti-COG3 and rabbit polyclonal affinity purified anti-COG8 primary and anti-mouse ATTO488 and anti-rabbit-Alexa647 secondary antibodies.

The superresolution 3D-SIM images were acquired using an Elyra PS.1 (Zeiss, Jena, Germany) equipped with an oil Plan-Apochromat objective lens (100 × , NA 1.46), EMCCD camera, and four lines of excitation lasers (405, 488, 561, and 642 nm) under the control of Zen 2012 software (Zeiss). The chromatic shift aberration of the system was calibrated and corrected by TetraSpeck beads (110 nm in diameter; Invitrogen). The pixel size was 40 nm. The *z*-step of image stacks was 80 nm. The intensity line profile of the Golgi region imaged by 3D**-**SIM and Pearson’s coefficient of colocalization was generated using ImageJ (National Institutes of Health, Bethesda, MD). Pearson’s coefficient was calculated using individual optical slices from the entire Golgi area of 10–18 cells.

Two-color STED images were acquired on the Leica TCS SP8 STED 3x microscope essentially as in Pellett *et al.*^51^. Two-color STED time series were obtained sequentially line by line by alternating the excitation lasers and the STED pulsed and gated lasers tuned to 775 and 592 nm for both Alexa 647 and ATTO488. A 100x/1.4 NA oil immersion objective lens (HC PL APO SC2, Leica Microsystems) was used for imaging. The pixel size was 20 nm.

### Transmission Electron Microscopy

Cells grown on collagen-coated sapphire discs were high pressure frozen using a Leica EM PACT2 high pressure freezer with rapid transfer system. Samples were transferred under liquid nitrogen to cryovials containing 2% OsO_4_/0.1% glutaraldehyde/1%H_2_O in acetone. Samples were freeze substituted in a Leica EM AFS2 freeze substitution and low temperature embedding system under the following schedule: −90 °C for 40 h, warm 3 °C/h to −60 °C, −60 °C for 8 h, warm 3 °C/h to −30 °C, −30 °C for 8 h, warm 3 °C/h to 0 °C. Following freeze substitution, samples were rinsed three times (10 minutes each) in acetone followed by tannic acid at 4 °C (1% tannic acid with 1% H2O in acetone) for 1 h. Samples were rinsed three times in acetone followed by 1 h osmium wash (1% OsO_4_/1% H_2_O solution in acetone) at 4 °C. Samples were rinsed three times in acetone and embedded in Araldite 502/Embed 812 resin (EMS).

Ultrathin sections were imaged at 80 kV on a FEI Technai G2 TF20 transmission electron microscope and images were acquired with a FEI Eagle 4kX USB Digital Camera

### GFP binding protein (GBP) Co-immunoprecipitation

DNA encoding GFP binding protein (GBP)^52^ (synthesized by Genscript) was inserted into pET24B vector. Recombinant His6x-tagged GBP was purified on Talon resin (Clontech), and then conjugated to high density glyoxal agarose beads (Agarose Bead Technologies). GBP beads were used for IP similar to agarose protein G beads. Cells were collected by trypsinization (HeLa) or by gentle resuspension (HEK293T), and lysed in 50 mM Tris, pH 7.4, 150 mM NaCl; 1% Triton X-100 supplemented with Halt protease inhibitor mixture (Thermo Scientific) and 1 mM PMSF for 1 hour on ice.

Cell lysate was spun at 20,000 × g and supernatant was added to 20–25 μL of 50% GBP-bead suspension. Bead-lysate mix was incubated for 2 h at room temperature while rotating. Unbound material was removed and the beads were washed three times in 0.05% Triton X-100 in PBS and eluted in 2X sample buffer to a total volume of 50 μL.

### Galanthus nivalis lectin labeling

Cells grown on 12 mm glass coverslips (#1, 0.17 mm thickness) were washed 3 times with DPBS and then fixed for 15 min with 1% PFA (16% aqueous stock solution; Electron Microscopy Sciences). After fixation, cells were incubated with 1% BSA in DPBS for 10 min. Cells were then incubated with *Galanthus Nivalus* lectin (GNL) labeled with AlexaFluor 647 (Invitrogen) diluted 1:500 in 0.1% BSA in PBS for 30 min. Cells were washed 5 times with DPBS and then fixed again with 4% PFA for 15 min. After second fixation cells were either mounted on glass microscope slides using Prolong^®^ Gold antifade reagent (Life Technologies) or were stained with antibodies following the IF protocol detailed above.

### Flow analysis using *Galanthus nivalis* lectin

COG1 KO, COG8 KO and COG1/COG8 double KO cells were grown in 3 wells of a 6 well plate along with wild-type HEK293T cells. 1 day after plating cells were transfected with Sialytransferase-RFP (a gift from J. Rothman lab, Yale University) and pCOG1-HA-GS-COG8-3Myc and/or hTEV in pLIK (a gift from the Puhl Lab, NIH) using Lipofectamine 2000 (Life Technologies). 48–72 hours after transfection cells were washed with DPBS, then resuspended in 0.1% BSA in DPBS then moved to a tube and placed on ice. After centrifugation at 800xg for 3 minutes supernatant was removed and cells were resuspended in 0.1% BSA in DPBS with *Galanthus Nivalus* lectin (GNL) labeled with AlexaFluor 647 (Life Technologies) diluted 1:500. After incubation cells were placed on ice in the dark for 30 minutes and resuspended in 0.1% BSA in DPBS with DAPI to distinguish live/dead cell populations. Cells were then analyzed using the LSRFortessa Flow Cytometer (BD Biosciences) or Attune NxT Acoustic Focusing cytometer (Life Technologies). Cells were gated based on shape, singlets, and DAPI signal. Transfected cells were also gated using ST-RFP signal to track transfection. Processing of data was done using NovoExpress software (ACEA Biosciences). The same protocol was followed for the COG1-GFP-GS-COG8-3myc construct, with the exception of gating for GFP instead of co-transfected ST-RFP.

### Isolation of whole COG and lobe B complex from HEK cells

HEK293T cells grown in five 10-cm dishes were transfected with MultiLabel vectors with Lipofectamine 2000 reagent (Life Technologies) as previously described^6^. (lobe B expression: GFP-hCOG5 + hCOG6/8-3Myc + hCOG7-3Myc; whole COG expression: GFP-mCOG3 + hCOG1/2/4-3Myc + hCOG6/8-3Myc + hCOG5/7-3Myc). Twenty four hours after transfection, cells were treated with 5 mM sodium butyrate. Cells were harvested 40 h after COG transfection and incubated in hypotonic lysis buffer (20 mM HEPES ,pH 7.5) supplemented with 1 mM PMSF and HALT protease inhibitor cocktail (Thermo Scientific) on ice for 10 min. Cells were further disrupted by passing through a 24-gauge needle 20 times. Then cell lysate was supplemented with 150 mM potassium acetate. After removing unbroken cells and nuclei by centrifugation at 2000xg for 10 min at 4 °C, 1% Octyl β-D-glucopyranoside was added into the supernatant which was centrifuged again at 20,000xg for 10 min at 4 °C.

The final cell supernatant (1 ml) was loaded onto 4 ml Sephacryl S-300 HR gel filtration (GF) column which was pre-equilibrated with GF buffer (20 mM HEPES, pH 7.5, 150 mM potassium acetate, 10% glycerol) supplemented with 0.1% Octyl β-D-glucopyranoside to remove small protein contaminants. Fractions enriched with COG complex were collected and kept on ice.

Recombinant His6x-tagged GBP was purified with TALON resin (Clontech) following a standard protocol. Fractions with highest concentrations of GBP were pooled together and extensively dialyzed against the dialysis buffer (25 mM HEPES-KOH, pH 7.4, 25 mM KCl, 2.5 mM magnesium acetate). TALON beads were incubated with purified recombinant GBP for 30 min at room temperature with rotating. Unbound proteins were removed by washing. Then *Sephacryl S-300 HR* GF fractions containing COG complex or lobe B were incubated with TALON-GBP beads for 30 min at room temperature. TALON-GBP beads were washed with GF buffer supplemented with 10 mM imidazole and then eluted with GF buffer supplemented with 350 mM imidazole. Fractions with COG complex were extensively dialyzed against the GBP dialysis buffer supplemented with 10% glycerol and 1 mM DTT at 4 °C overnight by using Micro Float-a-Lyzer (Spectrum labs, MWCO: 100 kD). After dialysis, proteins were frozen in liquid nitrogen and stored at −80 °C.

### Preparation of recombinant proteins

cDNAs encoding full-length human GS15 cytoplasmic domain (aa 1–86), cytoplasmic GS15 with deletion of N-terminal domain (Δ1–20), and deletion of SNARE domain (Δ21–86) were subcloned into pET28a (EMD Biosciences) with His6X tag fused at N-terminus and GST tag fused at C-terminus of the expressed protein. GS15 proteins were expressed in *E. coli* BL21(DE3) cells. pGEX4T-1 (GE Healthcare) was utilized to express recombinant GST protein in *E. coli* XL-10 Gold (Stratagene). All bacterial cultures were grown at 37 °C with shaking until they reached mid-log (OD_600_ = 0.5–0.6). Expression of proteins was induced by the addition of 0.2 mM isopropyl-1-thio-β-D-galactopyranoside (IPTG), and then the culture was grown over night at 22 °C. The cells were collected by centrifugation at 6000 g for 30 min at 4 °C and lysed in lysis buffer (50 mM Tris, 0.3 M NaCl, pH 8.0, 40 μg/ml lysozyme, 1 mM PMSF). His6X-tag conjugated GS15 proteins were purified by TALON metal affinity resin (Clontech) following a standard protocol. GST was purified by glutathione Sepharose 4B beads following a standard protocol. Fractions with highest concentrations of proteins were pooled together and extensively dialyzed against the buffer (25 mM HEPES, pH 7.4, 150 mM KCl, 1 mM DTT, 0.05% Tween-20). After dialysis, proteins were frozen in liquid nitrogen and stored at −80 °C.

### COG-GS15 binding assay

Glutathione Sepharose 4B beads were incubated with purified recombinant GST, full-length (FL) of GS15 cytoplasmic domain (aa 1–86), cytoplasmic GS15 with deletion of N-terminal domain (Δ1–20), and deletion of SNARE domain (Δ21–86) in the buffer (20 mM HEPES, pH 7.4, 150 mM KCl, 1 mM DTT, 0.05% Tween-20) for 2 hours at 4 °C with rotating. Unbound proteins were removed by extensive washing. Then whole COG complex or lobe B isolated from HEK cells were incubated with beads in the buffer (25 mM HEPES-KOH, pH 7.4, 25 mM KCl, 2.5 mM magnesium acetate, 1 mM DTT, 0.05% Tween-20) for 1 hour at room temperature with rotating. After washing, the remaining beads-bound material was eluted with 2X SDS-PAGE sample buffer with heating for 10 min at 70 °C and resolved with SDS–PAGE and WB.

### SDS–PAGE and Western blotting (WB)

SDS–PAGE and WB were performed as described previously^12^. Blots were incubated with primary rabbit polyclonal antibodies and mouse monoclonal antibodies, followed by incubation with appropriate secondary antibodies conjugated with IRDye 680 or IRDye 800 dyes (LI-COR). Blots were scanned and analyzed with an Odyssey IR imaging system (LI-COR).

### Multiple COG expression assay for co-IP

HEK293T cells were transfected with multi-expression vectors as previously described^6^ for 36 h using Lipofectamine 2000 (Life Technologies) reagent. (lobe A expression: hCOG1/2/4-3Myc + mCOG3-3Myc; lobe B expression: hCOG6/8-3Myc + hCOG5/7-3Myc; lobe A + lobe B; hCOG1/2/4-3Myc + mCOG3-3Myc + hCOG6/8-3Myc + hCOG5/7-3Myc). After 18 h of COG-Myc expression cells were transfected again with GFP expression plasmids using Lipofectamine 2000 (Life Technologies) reagent. Cells were harvested 36 h after initial COG transfection and processed for IP.

### Statistical analysis

Results shown are mean ± standard deviation based on a sample (STDEV.S). Statistical testing was performed using Student’s t-test. *p < 0.05; **p < 0.01; ***p < 0.001. For immunofluorescence microscopy analysis, the number of cells was greater than 10. The number of experiments was greater than three for each quantification.

## Additional Information

**How to cite this article**: Willett, R. *et al.* COG lobe B sub-complex engages v-SNARE GS15 and functions via regulated interaction with lobe A sub-complex. *Sci. Rep.* **6**, 29139; doi: 10.1038/srep29139 (2016).

## Supplementary Material

Supplementary Information

## Figures and Tables

**Table 1:** Plasmids used in this study.

Plasmid name	Description	Source
hCOG1 1-702-3Myc	Human COG1(aa1-702) in pCMV-3Myc	This study
hCOG1-3Myc	Human Cog1siRES in pCMV-3Myc	Willett *et al.*^6^
mCOG3-3Myc	Mouse COG3 in pCMV-3Myc	Willett *et al.*^6^
hCOG7-3Myc	Human COG7 in pCMV-3Myc	Ha *et al.*^15^
hCOG6-3Myc/hCOG8-3Myc	Human COG6-3Myc and COG8-3Myc in MultiLabel vectors (ATG:biosynthetics GmbH)	Willett *et al.*^6^
hCOG5-3Myc/hCOG7-3Myc	Human COG5-3Myc and COG7-3Myc in MultiLabel vectors (ATG:biosynthetics GmbH)	Willett *et al.*^6^
hCOG2-Strep-3Myc/hCOG4-3Myc/hCOG1-3Myc	Human COG1-3Myc, COG2Streptag-3Myc, and COG4-3Myc in MultiLabel vectors (ATG:biosynthetics GmbH)	Willett *et al.*^6^
hCOG1-(HA-GS-) or (GFP-GS-) TEV-hCOG8-3Myc	Human COG1-siRES, COG8-siRES and TEV protease recognition sequence in pCMV-3Myc	This study
hCOG1-TEV-3Myc	Human COG1-siRES and TEV protease recognition sequence in pCMV-3Myc	This study
hCOG8sir-mCh-ActA	Human COG8siRES in pmCherry-ActA	Willett *et al.*^22^
hCOG8sir-GFP-ActA	Human COG8siRES in pGFP-ActA	This study
hCOG8sir 1–406 GFP-ActA	Human COG8siRES (aa1–406) in pGFP-ActA	This study
COG8sir 1–136 GFP-ActA	Human COG8siRES (aa1–136) in pGFP-ActA	This study
hCOG8sir Δ8–406 GFP-ActA	Human COG8siRES (aaΔ8–406) in pGFP-ActA	This study
hCOG8sir Δ8-184-3Myc	Human COG8siRES (aaΔ8-184) in pCMV-3Myc	Willett *et al.*^22^
GFP-hGS15	Human GS15 in pEGFP-C1	Shestakova *et al.*^17^
GFP-P115	P115 in pEGFP-C2	E. Sztul
GFP-ActA		A. Linstedt
GFP-hCOG6	Human COG6 in pGFP-C1	Pokrovskaya *et al.*^9^
GFP-mCOG3	Mouse COG3 in pEGFP-C1	Zolov *et al.*^40^
GFP-hCOG5	Human COG5 in pEGFP-C1	Ha *et al.*^15^
GFP	pEGFP-C1	
His-hGS15-GST	Human GS15 cytoplasmic domain (aa 1–86) with N-terminal polyhistidine tag and C-terminal GST tag in pET28a	This study
GST	pGEX4T-1	
