# Supplementary material for: COG lobe B sub-complex engages v-SNARE GS15 and functions via regulated interaction with lobe A sub-complex
Source: Sci Rep. 2016 Jul 7;6:29139. doi: 10.1038/srep29139 (PMC4935880; doi:10.1038/srep29139)
Supplement: Supplementary Information [file srep29139-s1.pdf]

Supplementary Information

June 1, 2016

COG lobe B sub-complex engages v-SNARE GS15 and functions via regulated interaction with lobe A sub-complex.

Rose Willett[1], Jessica Bailey Blackburn[1], Leslie Climer[1], Irina Pokrovskaya[1], Tetyana Kudlyk[1], Wei Wang[1] and Vladimir Lupashin[1]*

[1]Department of Physiology and Biophysics, UAMS, Little Rock, AR

***Corresponding author:**

Dr. Vladimir Lupashin

Department of Physiology and Biophysics

University of Arkansas for Medical Sciences

Biomed 261-2, Mail slot 505

4301 West Markham Street, Little Rock, AR 72205, USA.

tel 501-603-1170 ; fax 501-686-8167

vvlupashin@uams.edu

Supplementary Information

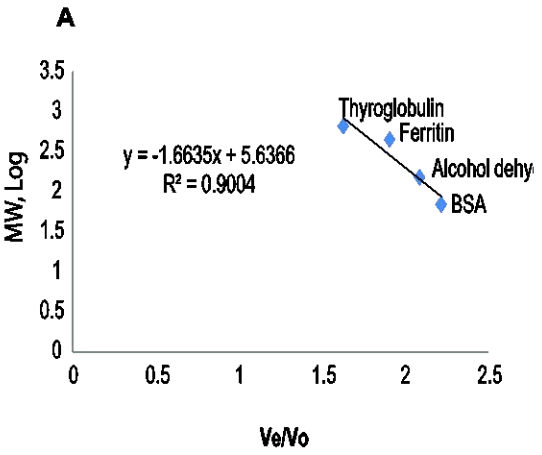

B

Protein	MW (kDa)	Log(MW)	Ve	Vo	Ve/Vo	Ve peak fraction #
Thyroglobulin	660	2.8195	12.403	7.593	1.63	8
Ferritin	450	2.6532	14.475	7.593	1.91	12
Alcohol dehydrogenase	150	2.176	15.88	7.593	2.09	15
BSA	69	1.8388	16.523	7.593	2.22	16

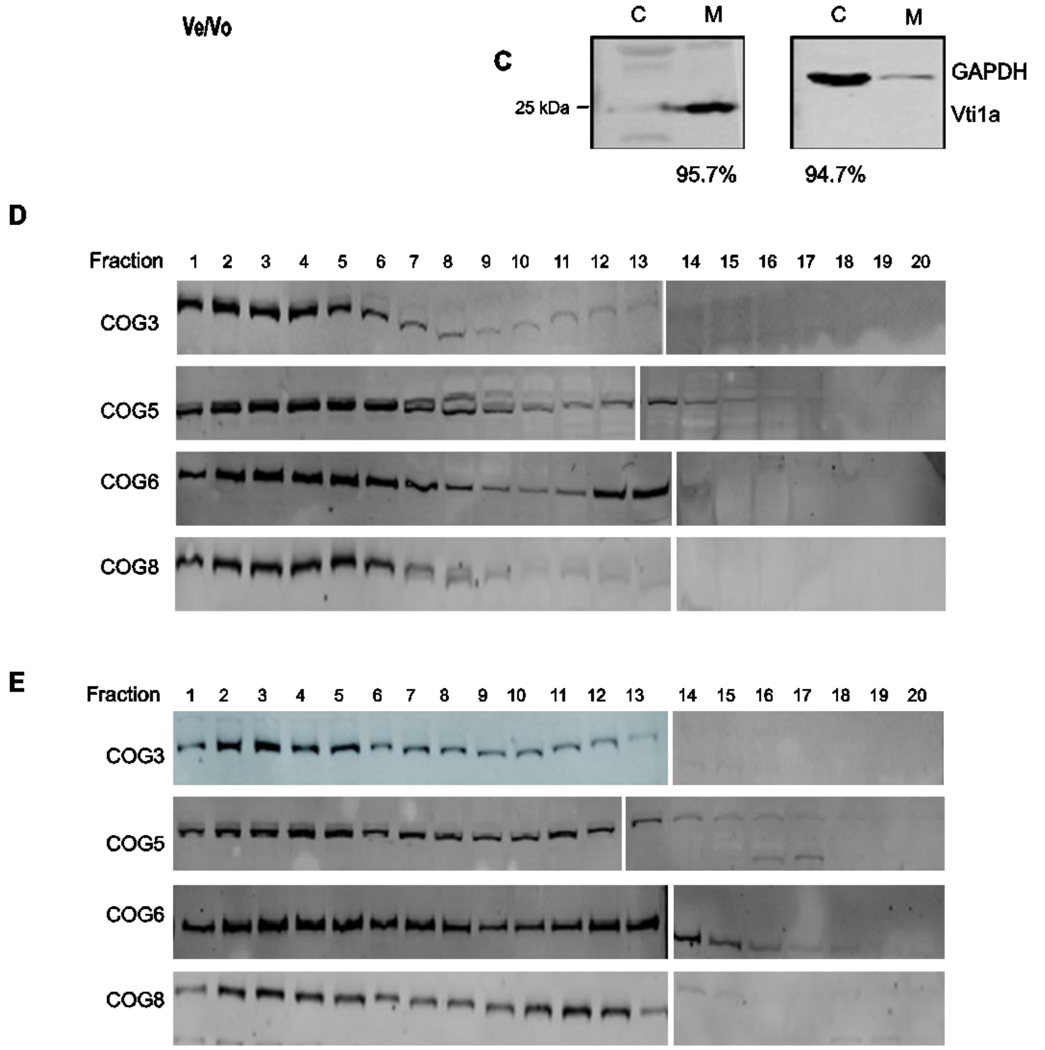

Supplemental Figure S1: Gel filtration of COG subcomplexes. Superose 6 10/300GL was equilibrated in column buffer (125mM Potassium Acetate 25mM HEPES

pH7.2). (**A**) Ferritin from equine spleen (Sigma, Type I, saline solution), Alcohol dehydrogenase (Worthington Biochemical Corporation), and Bovine serum albumin (RPI) were diluted with column buffer plus 0.1% Tween 20 to 10mg/ml and 100ul were run on a Superose 6 10/300GL column at .4ml/min both together and separately. The void volume (Vo) at ~7.5ml was used with the elution volume (Ve) to generate a protein standard curve to calculate the size of eluted proteins. $y = -1.6635x + 5.6366$ $R^2 = 0.9004$. (**B**) Fraction volumes of protein standards for calculating protein standard curve. (**C**) Distribution of membrane (M) and cytosol (C) material. HeLa cells were lysed by vortexing with 700ul .5mm glass beads and then cell lysate was separated by differential centrifugation. Separation of material was ~95%. (**D, E**) Western blots. HeLa cell cytosol (**D**) or Triton-X100 soluble membranes (**E**) were loaded onto a Superose 6 10/300GL and 0.5mL fractions were collected. Fractions were concentrated by TCA precipitation, separated on a SDS-PAGE gel, and blotted with antibodies against COG3, COG5, COG6, and COG8.

A

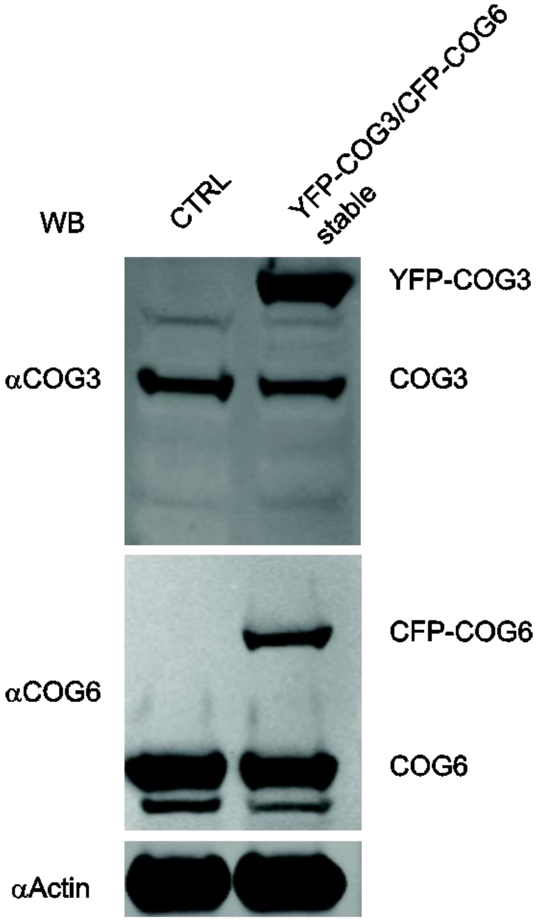

WB
CTRL
YFP-COG3/CFP-COG6 stable
αCOG3
YFP-COG3
COG3
αCOG6
CFP-COG6
COG6
αActin

B

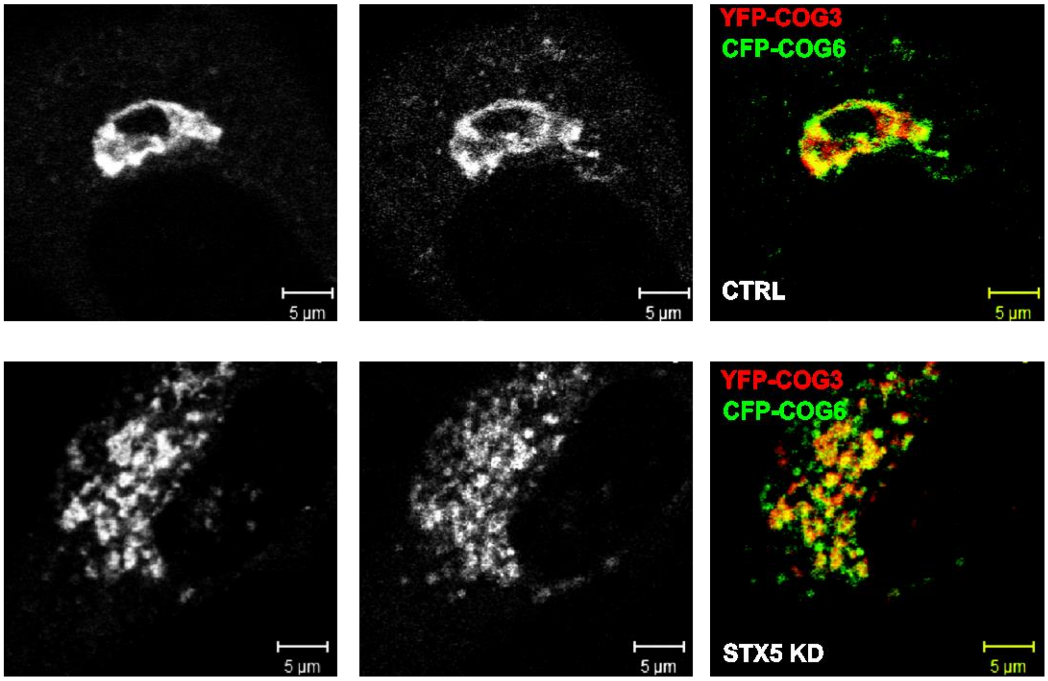

YFP-COG3
CFP-COG6
CTRL
STX5 KD
5 µm

Supplemental Figure S2: Fluorescently tagged COG subunits are expressed in stable HeLa cells at nearly endogenous level. (**A**) Total proteins from stably transfected YFP-COG3/CFP-COG6 HeLa cells were separated on an SDS–PAGE gel, and blotted with antibodies against COG3 (upper panel), COG6 (middle panel) and actin (lower panel). Blots were scanned with an Odyssey IR imaging system (LI-COR). (**B**) HeLa YFP-COG3/CFP-COG6 stable cells or same cells depleted for Golgi t-SNARE STX5 were fixed and analyzed by IF. Bar, 5 μm.

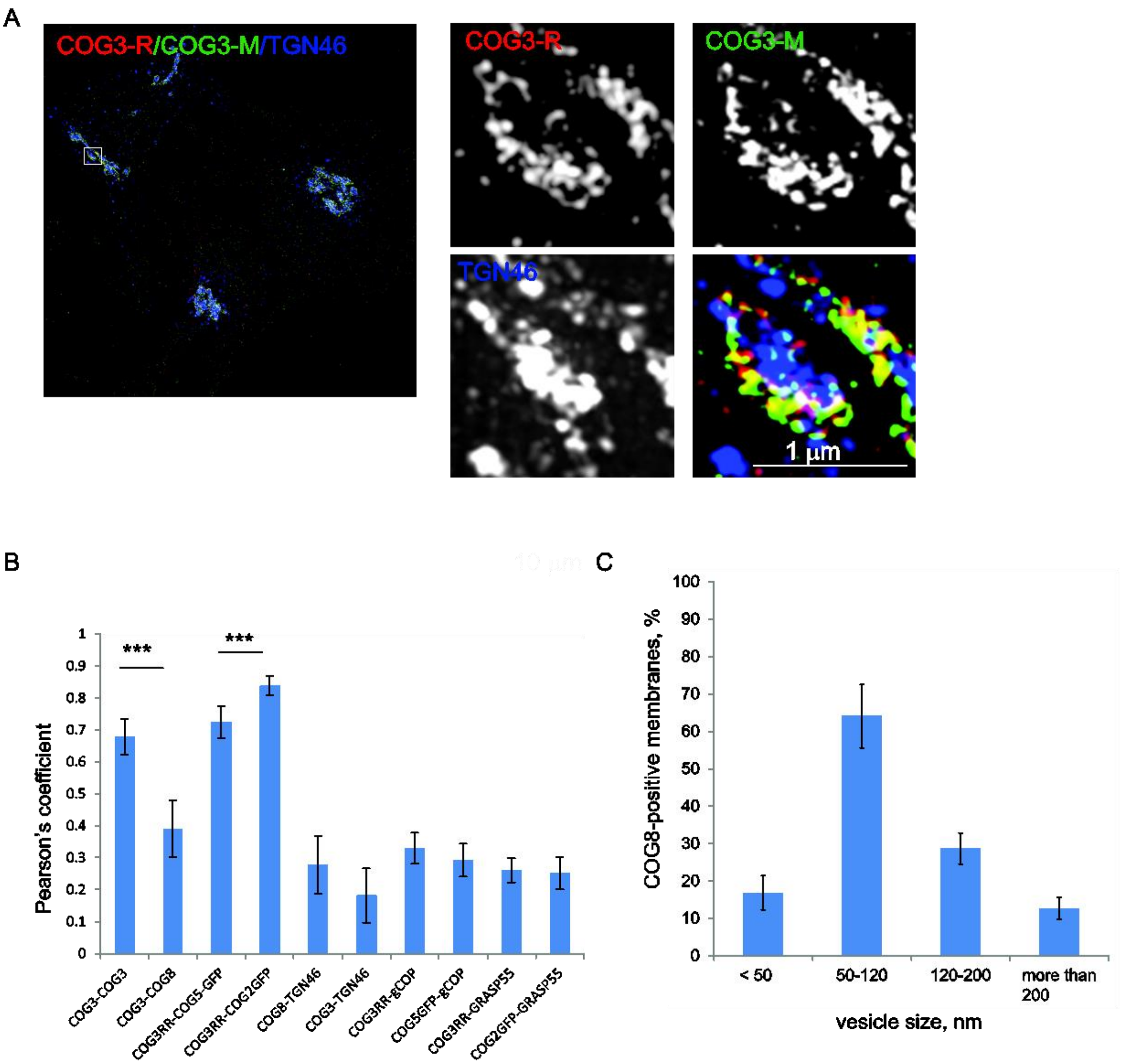

Supplemental Figure S3: Super-resolution microscopy. (**A**) HeLa cells were stained for rabbit anti-COG3-Alexa 555, mouse monoclonal anti-COG3-Alexa 488 and sheep-TGN46-Alexa 647 and imaged using the Zeiss ELYRA S1 (SR-SIM) (**B**) Pearson's co-localization coefficients for 3D SIM superresolution images. Pearson's coefficient was calculated using individual optical slices from the entire Golgi area of 10-18 cells. (**C**) Size distribution of COG8-positive Golgi vesicles in 3D SIM images. 11 individual Golgi slices stained with anti-COG8 antibodies were analyzed using ImageJ program. COG8 positive membranes were clustered in four groups based on their size: very small

membranes/protein complexes (less than 50 nm in size), intra-Golgi vesicles (50-120 nm), vesicle clusters (120-200 nm) and large Golgi membranes (more than 200 mn in size).

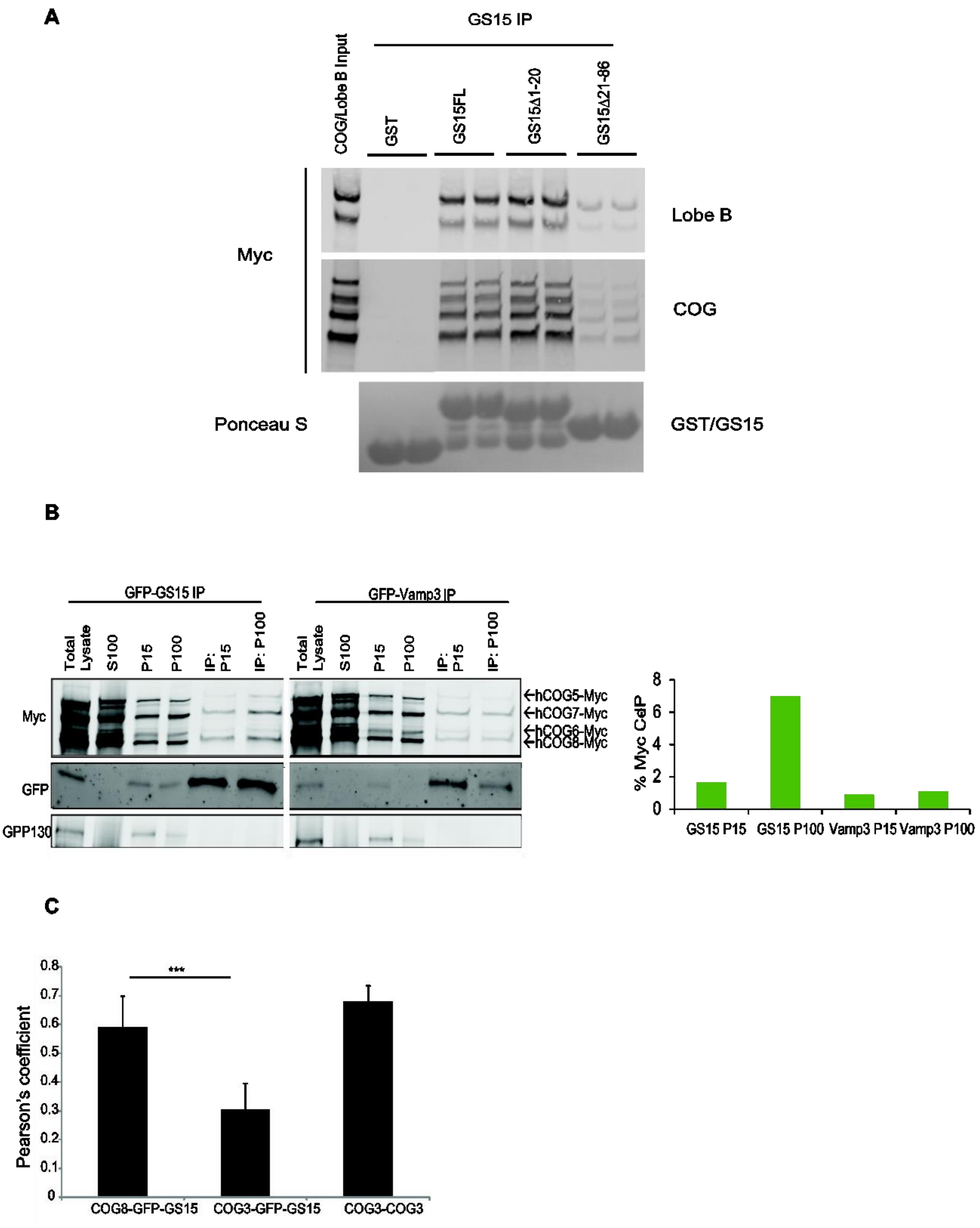

Supplemental Figure S4: COG sub-complex lobe B preferentially interacts with GS15 on vesicles. (**A**) COG-GS15 binding assay. Glutathione beads were incubated

with recombinant GST, GST-tagged full-length (FL) of GS15 cytoplasmic domain (aa 1-86), GS15 with deletion of N-terminal domain (Δ1-20), and deletion of SNARE domain (Δ21-86). Unbound proteins were removed by extensive washing. Whole COG complex or lobe B isolated from HEK cells were incubated with beads for 1 hour at room temperature. After wash, the remaining beads-bound material was eluted and resolved on an SDS–PAGE gel along with total whole COG or lobe B input. The separated proteins were transferred to nitrocellulose membrane and beads bound GS15 was visualized by Ponceau S staining. Blots were then probed with the antibody against Myc and scanned with an Odyssey IR imaging system (LI-COR). **(B)** HEK 293T cells were transiently transfected with COG-Myc multi-expression plasmids that express the same triple-myc epitope tag allowing for precise quantification of interactions (lobe B expression: hCOG6/8-3Myc + hCOG5/7-3Myc) and 24 h later with GFP-GS15 or GFP-Vamp3. 24 h post second transfection, cells were collected and fractionated into large membrane (P15), small membrane (P100), and cytosol (S100) by differential centrifugation. The membrane pellets were resuspended in lysis buffer and then incubated with GBP-beads. Immunoprecipitates, along with 10% of total input, were separated on an SDS–PAGE gel, transferred to nitrocellulose membrane and probed with antibodies against Myc (upper panel) and GFP (middle panel) and GPP130 (lower panel). Blots were scanned with an Odyssey IR imaging system (LI-COR) and analyzed using Image Studio (LI-COR) fluorescence quantitative imaging. Co-immunoprecipitation efficiency values were generated by calculating the relative density of the co-IP Myc signal relative to the density of the input Myc signal. Note that lobe B interacts preferentially with GFP-GS15 on vesicle enriched membrane fraction (P100)

and that Vamp3 shows no preferential interaction. (**C**) Pearson's co-localization coefficients for superresolution images in Figure 4G.

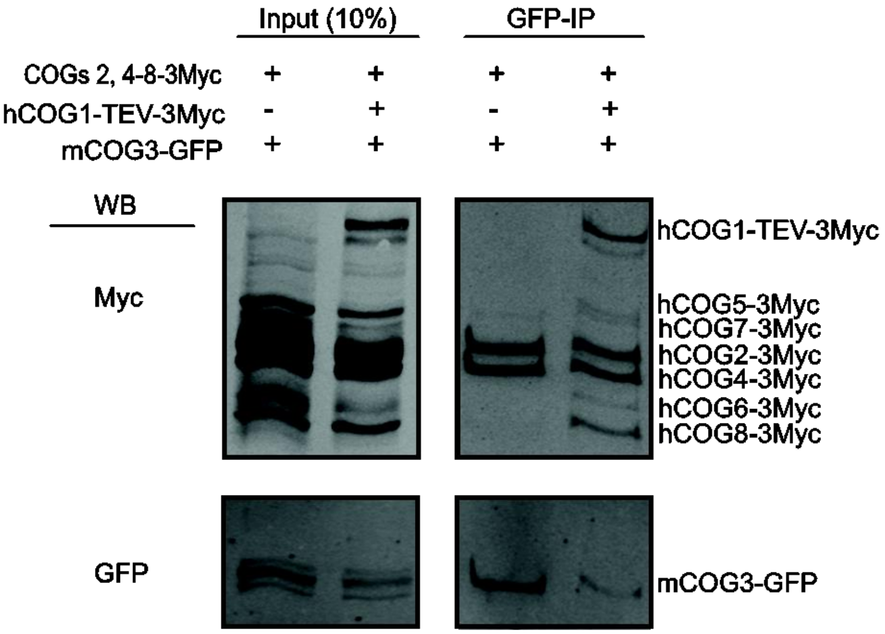

Supplemental Figure S5: COG1 and COG8 interaction is essential for lobe A-lobe B sub-complex bridging. HEK293T stable COG1 KO cells were transfected with plasmids encoding mCOG3-GFP, hCOG2-3Myc, hCOG4-3Myc, hCOG6/hCOG8-3Myc, hCOG5/hCOG7-3Myc with and without expression of hCOG1-TEV-3Myc. 24h after transfection cells were collected and lysed in IP buffer and then incubated with GBP-beads. Immunoprecipitates, along with 10% of total input, were separated by SDS–PAGE and blotted with antibodies against Myc (upper panel) and GFP (lower panel). mCOG3-GFP IP was only capable of recovering hCOG8-3Myc upon expression of COG1, indicating that lobe A-lobe B interaction is directly facilitated by the COG1-COG8 protein-protein interaction.

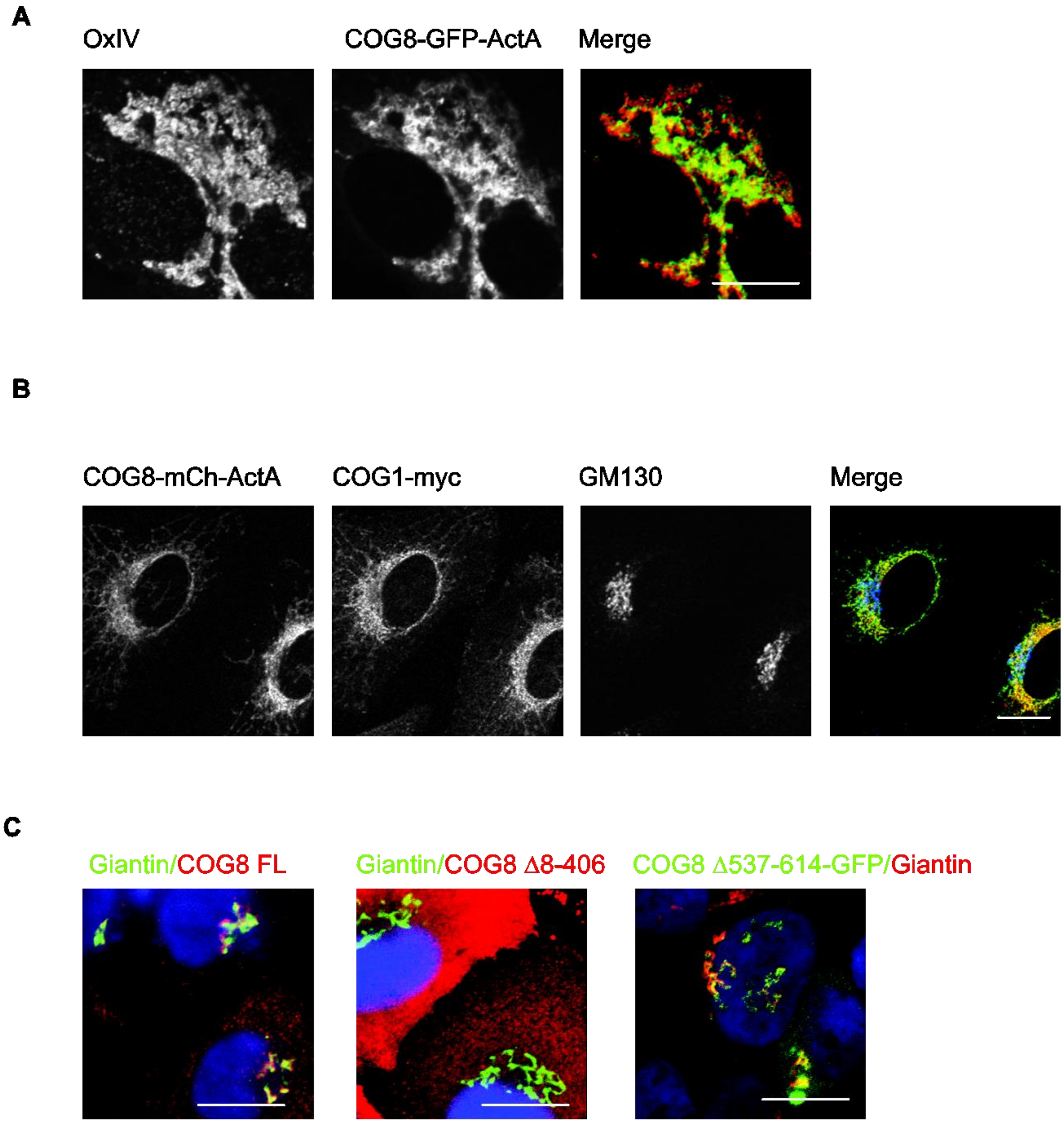

Supplemental Figure S6: Mitochondrial targeted COG8 recruits full-lengh COG1 in HeLa cells. (**A**) COG8-GFP-ActA co-localizes with mitochondria decorated with anti-OxIV antibodies. (**B**) COG8-mCherry-ActA recruits COG1-myc to mitochondria. (C) Full length COG8-myc of COG8 Δ537-614-GFP were co-localized with Golgi marker Giantin in transiently transfected HeLa cells while COG8 Δ8-406 failed to localize to the Golgi.

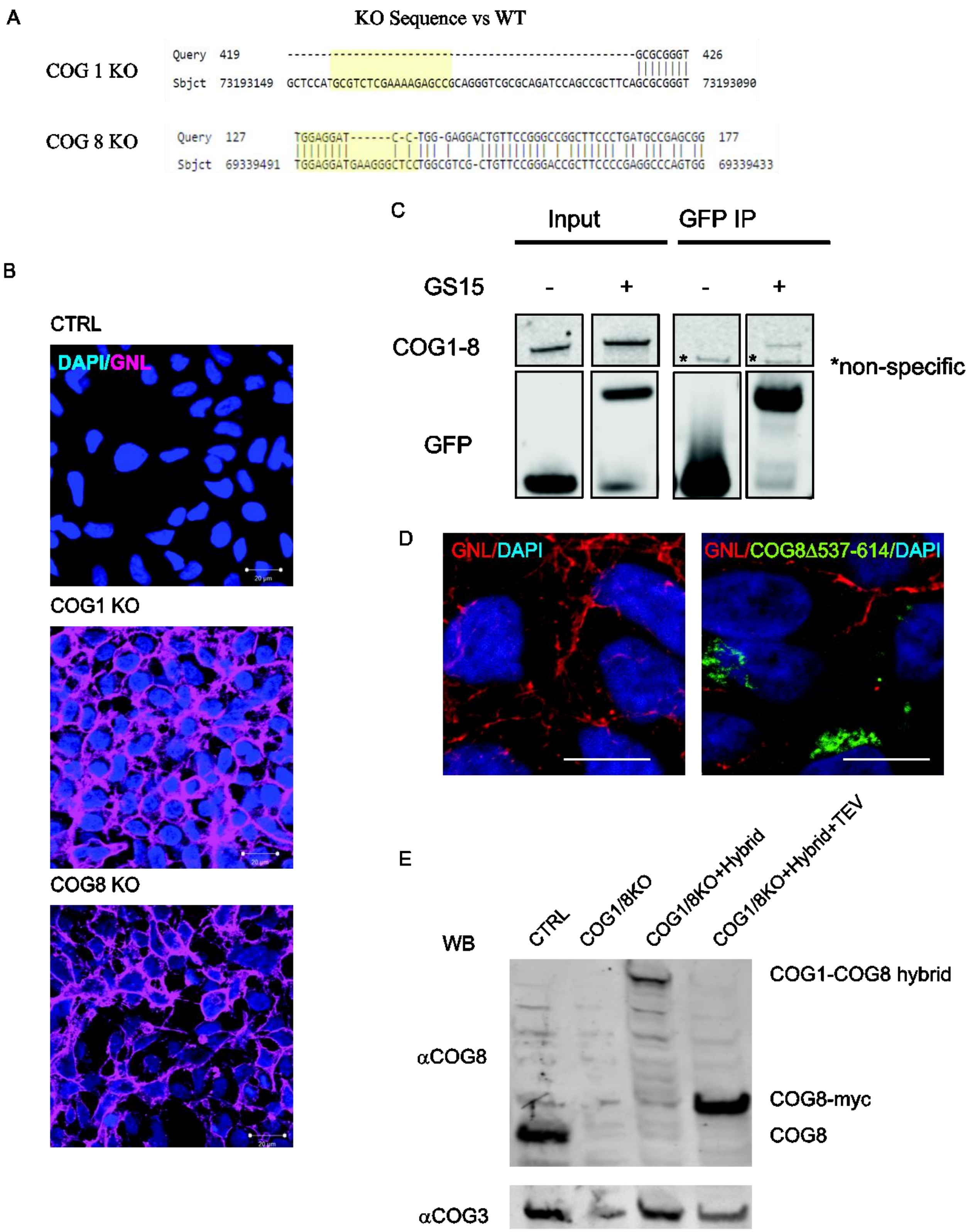
A
KO Sequence vs WT
COG 1 KO
Query 419 ---GCGCGGGT 426
Sbjct 73193149 GCTCCATGCGTCTCGAAAAGAGCCGCAGGGTCGCGCAGATCCAGCCGCTTCAGCGCGGGT 73193090
COG 8 KO
Query 127 TGGAGGAT------C-C-TGG-GAGGACTGTTCCGGGCCGGCTTCCCTGATGCCGAGCGG 177
Sbjct 69339491 TGGAGGATGAAGGGCTCCTGGCGTCG-CTGTTCCGGGACCGCTTCCCCGAGGCCCAGTGG 69339433
B
CTRL
DAPI/GNL
COG1 KO
COG8 KO
C
Input
GFP IP
GS15
-
+
-
+
COG1-8
GFP
*non-specific
D
GNL/DAPI
GNL/COG8Δ537-614/DAPI
E
WB
CTRL
COG1/8KO
COG1/8KO+Hybrid
COG1/8KO+Hybrid+TEV
αCOG8
COG1-COG8 hybrid
COG8-myc
COG8
αCOG3

Supplemental Figure S7: Characterization of COG KO cells and interaction between COG1-COG8 hybrid with GFP-GS15. (**A**) DNA sequence alignments for chromosomal sequences amplified from COG KO (upper line) and control HEK293T cells. (**B**) Plasma membrane of COGKO cells are bound with mannose-specific GNL-647 lectin. Control, COG1 KO and COG8 KO HEK293T cells grown on collagen-coated gall coverslips were fixed in 1% PFA, stained with GNP-Alexa-647 and analyzed by confocal microscopy. (C) COG1-COG8 hybrid is precipitated in GFP-GS15 IP. HEK293T COG1/COG8 double KO cells were co-transfected with plasmids encoding GFP + COG1-HA-GS-COG8 hybrid or GFP-GS15 + COG1- HA-GS-COG8 hybrid. 24 hours after transfection cells were collected and lysed in IP buffer and then incubated with GBP-beads. Immunoprecipitates, along with 10% of total input, were separated by SDS–PAGE and blotted with antibodies against Myc (upper panel) and GFP (lower panel). (D) COG8 KO cells were either untransfected or transfected with plasmids encoding either COG8 Δ537-614-GFP. 72 hours after transfection cells were fixed and surface labeled with GNL-647. (E) COG1- HA-GS-COG8 hybrid expressed in near endogenous level. HEK293T cells, COG1/COG8 double KO cells, COG1/COG8 double KO cells transfected with COG1- HA-GS-COG8 hybrid or co-transfected with COG1-HA-GS-COG8 hybrid and plasmid encoding TEV protease were lysed and analyzed with WB using anti-COG8 (upper panel) and anti-COG3 antibodies.

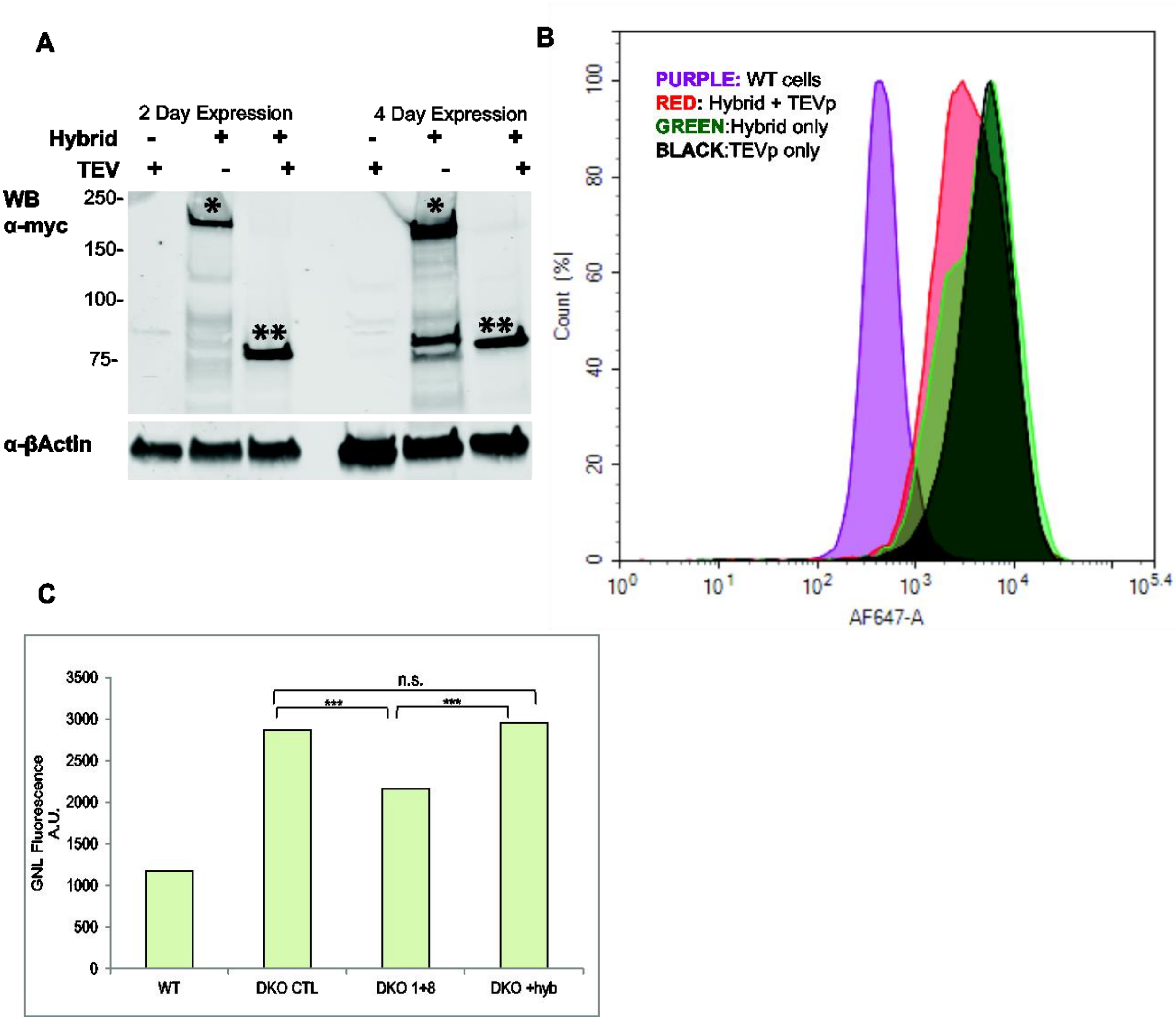

Supplemental Figure S8: (A) Lysates of HEK293T cells transfected with the COG1-HA-GS-COG8 Hybrid, hTEV or both were prepared 2 or 4 days post transfection. Lysates were separated on an SDS-PAGE gel and blotted with antibodies against Myc (upper panel) and β-actin (lower panel). * denotes full length COG1- HA-GS-COG8 Hybrid. ** Denotes hTEV cleaved product. (B) Flow cytometry analyzing GNL-647 binding to COG1/COG8 KO cells analyzed after 48hrs of transfection with COG1- HA-GS-COG8 hybrid (green), hTEV (black), or both (red). WT cells stained with GNL-647

included as a negative control (purple). (C) Flow cytometry results analyzing GNL-647 binding to COG1/COG8 KO cells analyzed after 48hrs of transfection with COG1-GFP-COG8 hybrid or COG1-GFP and COG8-GFP individually. Histogram shows median fluorescence of GNL-647 for GFP+ populations. *** indicates $p<0.001$. Note the apparent partial rescue of glycosylation defect observed in COG1/COG8 KO cells co-transfected with COG1-GFP-COG8 hybrid and TEV protease plasmids was due to a short expression time and a relatively slow renewal rate of plasma membrane glycoconjugates.
